# Metagenomic evidence for taxonomic dysbiosis and functional imbalance in the gastrointestinal tracts of children with cystic fibrosis

**DOI:** 10.1038/srep22493

**Published:** 2016-03-04

**Authors:** Ohad Manor, Roie Levy, Christopher E. Pope, Hillary S. Hayden, Mitchell J. Brittnacher, Rogan Carr, Matthew C. Radey, Kyle R. Hager, Sonya L. Heltshe, Bonnie W. Ramsey, Samuel I. Miller, Lucas R. Hoffman, Elhanan Borenstein

**Affiliations:** 1 Department of Genome Sciences, University of Washington, Seattle, US; 2 Department of Pediatrics, University of Washington, Seattle, US; 3 Department of Microbiology, University of Washington, Seattle, US; 4 Department of Medicine, University of Washington, Seattle, US; 5 Department of Computer Science and Engineering, University of Washington, Seattle, US; 6 Seattle Children’s Hospital, Washington, US; 7 Santa Fe Institute, New Mexico, US

## Abstract

Cystic fibrosis (CF) results in inflammation, malabsorption of fats and other nutrients, and obstruction in the gastrointestinal (GI) tract, yet the mechanisms linking these disease manifestations to microbiome composition remain largely unexplored. Here we used metagenomic analysis to systematically characterize fecal microbiomes of children with and without CF, demonstrating marked CF-associated taxonomic dysbiosis and functional imbalance. We further showed that these taxonomic and functional shifts were especially pronounced in young children with CF and diminished with age. Importantly, the resulting dysbiotic microbiomes had significantly altered capacities for lipid metabolism, including decreased capacity for overall fatty acid biosynthesis and increased capacity for degrading anti-inflammatory short-chain fatty acids. Notably, these functional differences correlated with fecal measures of fat malabsorption and inflammation. Combined, these results suggest that enteric fat abundance selects for pro-inflammatory GI microbiota in young children with CF, offering novel strategies for improving the health of children with CF-associated fat malabsorption.

People with the genetic disease cystic fibrosis (CF) have dysfunction in multiple organs with epithelial-lined lumens. Among these individuals, disease of the respiratory and gastrointestinal (GI) tracts are the most important determinants of quality and length of life^1^. The manifestations of CF GI disease begin early in life and include maldigestion and malabsorption of specific nutrients, particularly proteins, fats, and fat-soluble vitamins. This malabsorption leads not only to malnutrition, but also to intestinal obstruction and altered transit times of luminal contents through the GI tract^2^. Therefore, the CF GI tract has an abnormal physicochemical environment that could select for different GI microbiota.

In support of this hypothesis, we recently identified a fecal dysbiosis among children with CF characterized by a strikingly high abundance of *Escherichia coli* that was most marked in samples from younger subjects. We further showed that the magnitude of this *E. coli* dysbiosis correlated with a fecal measure of inflammation (calprotectin) as well as with fecal fat content^3^. However, a more systematic profiling of the CF microbiome could elucidate additional relationships between dysbiosis, intestinal inflammation, and luminal fat content.

In this study, we therefore set out to comprehensively analyze metagenomic data from this fecal sample collection, to fully characterize the taxonomic and functional composition of these metagenomes, and to leverage the longitudinal nature of the sample collection in order to better define CF-associated taxonomic dysbioses and functional imbalances, their temporal dynamics, and their links to inflammation and fat content. We hypothesized that taxonomic dysbiosis in the fecal microbiota of children with CF would lead to imbalances in the fecal abundance of microbial genes involved in the metabolism of fat or products related to host inflammation. Our results support a model of CF GI dysbiosis and dysfunction in which the malabsorption of dietary fat selects for a pro-inflammatory enteral microbiome.

## Methods

### Subjects and samples

The samples and source subjects for this analysis were collected as part of a prior study comparing the fecal microbiota of children aged <3 years old with and without CF. This study was approved by the Seattle Children’s Hospital Institutional Review Board, all procedures were carried out in accordance with the approved guidelines, and informed consent was obtained for all subjects. Subject inclusion/exclusion criteria, as well as demographic information, have been previously described^3^. The current analysis included not only the specimens described in our prior publication^3^, but additional specimens collected from the same subjects after our earlier analysis has begun. The final sample set (after removing low-coverage samples as described below) comprised 104 fecal samples from 14 children with CF (sampled between the ages of 15 days to 5 years) and 12 children without CF (sampled between the ages of 55 days to 3.5 years), and with between 2–5 samples per subject collected over an approximately one year period. In several analyses described here, we additionally binned samples by age group (e.g., first, second, or third year of life) to further control for potential age-related differences in the microbiome. Detailed information regarding the included specimens and source patients are provided in Supplementary Table S1.

### Fecal fat and calprotectin analyses

Fecal fat content was measured by the acid steatocrit method, performed as previously described for each sample in the collection^3^. Calprotectin–a product of neutrophils – was used as a fecal measure of inflammation and was defined using an FDA-approved enzyme-linked immunosorbent assay as previously described^3^.

### Metagenomic sequencing

Sample processing and DNA extraction were performed as previously described^3^. Briefly, sequencing data for this study resulted from Illumina HiSeq-2000 sequencing using the Nextera platform. The Human Microbiome Project (HMP) protocol was used for processing reads^4^,^5^. Specifically, BMTagger was used to remove human reads. Duplicates were removed using the HMP documented protocol. Runs for which a pair failed were not duplicate-filtered. Reads were quality-trimmed using HMP scripts, modified to work with single-end runs. Reads shorter than 60 bases after quality trimming were removed. Four samples that had less than 10 M reads after filtering human reads were also removed from the analysis. This process resulted in a total of 104 samples with an average of 58 million reads per sample.

### Taxonomic profiling and analysis

The taxonomic composition of each sample was defined using metagenomic phylogenetic analysis (MetaPhlAn^6^; version 1.7.3). To examine variation in taxonomic profiles across samples, a principal component analysis (PCA) was performed. Differentially abundant taxa in CF versus non-CF samples were identified using the Wilcoxon rank-sum test with false discovery rate (FDR) <0.1. To examine the role of *E. coli* in CF vs. non-CF samples, we performed several analyses using both the original taxonomic profiles and taxonomic profiles in which *E. coli* was excluded and the abundances of the remaining species were renormalized within each sample. In addition, we confirmed that our results held when excluding samples that were taken from children who had been administered antibiotics in the prior 60 days, and again when excluding samples from children who were breastfed at sampling.

### Functional annotation of metagenomic reads

To determine the presence and relative abundance of genes in each metagenomic sample, reads were mapped to the Kyoto Encyclopedia of Genes and Genomes (KEGG). Specifically, each sequencing read was aligned to a peptide database containing the peptide sequences from all annotated KEGG organisms (KEGG^7^; v. 67.0, July 15^th^, 2013 weekly release) using mBLASTx with standard parameters and accepting all matches with an E-value < 1 (in accordance with HMP protocol^4^,^5^). Each read was then annotated according to the KEGG Orthology groups (KOs) associated with the identified alignments using the ‘top gene’ approach that was previously described and carefully validated^8^. Notably, while significantly fewer KOs were identified in CF versus non-CF samples on average (rank-sum p < 0.03), when adjusted for the number of reads per sample (which were on average lower in CF samples), this difference became non-significant.

To streamline the analysis, we analyzed the data at the level of KEGG functional pathways and modules by summing the relative abundances of all KOs associated with each pathway or module. Pathways and modules were further filtered to verify that downstream analysis considers only bacterial pathways/modules. Specifically, a pathway (module) was included in our analysis only if at least 1% of bacterial genomes in KEGG contained at least 1 KO from that pathway (module), and if these bacterial genomes contained at least 5% (20%) of the KOs in the pathway (module) on average. Using this criterion resulted in a list of 146 and 409 bacterial pathways and modules, respectively.

### Comparative statistical analysis of functional profiles

For each pathway/module, CF samples were compared to non-CF samples using the Wilcoxon rank-sum test including a multiple comparisons correction using a 5% false discovery rate (FDR) threshold for both pathways and modules. Considering the relatively small number of samples available, several analyses were done by comparing all CF samples to all non-CF samples, ignoring age and subject identity. In additional analyses, samples were further binned by age group, to better control for subject age. For each pathway/module, the median number of non-zero KOs (i.e., KOs identified) across all samples was calculated, and only pathways and modules with more than 20 or 5 non-zero KOs, respectively, were considered. PCA was used to explore variation in functional profiles across samples. To further examine the patterns observed in this PCA, we additionally applied MUSiCC, a novel marker genes-based normalization scheme for accurate profiling of gene abundances in the microbiome^9^. In addition, we confirmed that our results held when excluding samples that were taken from children who had been administered antibiotics prior to sampling, both within 30 and 60 days, and when excluding samples from children who were breastfed at the time of sampling. We further confirmed that our main findings held when restricting our dataset to include only a single sample from each subject (from the first year of life), to verify that our results were not biased by the presence of multiple samples per individual.

### BWA-based analysis of the impact of *E. coli* on fatty acid biosynthesis pathway

To verify that the observed depletion of the fatty acid biosynthesis pathway is not solely the result of increased *E. coli* abundance in CF samples, we re-mapped the reads from all samples to a set of genome clusters using BWA^10^, following the method described in Greenblum *et al*.^11^. We analyzed the relative abundance of the fatty acid biosynthesis pathway using this alternative mapping method, confirming that it reproduced results obtained with the original BLASTx-based mapping (specifically, the depletion of this pathway in CF samples; P = 2.9e–05, Wilcoxon rank-sum test). We then removed all reads that mapped to the *E. coli* genome cluster and applied the same analysis, confirming that the fatty acid biosynthesis pathway was still significantly depleted in CF samples (P = 0.039).

### Creation of butyrate and propionate catabolism modules

To create functional modules representing butyrate catabolism and non-catabolism, a list of 8 enzymes from the butyrate metabolism pathway found to be associated with butyrate catabolism^12^,^13^ was compiled based on a detailed literature survey. The 14 KOs corresponding to these 8 enzymes were defined as the “butyrate catabolism” module, while the other 50 KOs representing enzymes in the butyrate metabolism pathway were defined as the “butyrate non-catabolism” module. Removing *bcd* (K00248) or both *bcd* and *atoA-atoD* (K01034, K01035), for which literature support was weaker, from the “butyrate catabolism” module did not qualitatively change the results reported below. We similarly partitioned the propionate metabolism pathway into propionate catabolism (19 KOs) and propionate non-catabolism (43 KOs) modules^14^,^15^.

## Results

### Taxonomic analysis of pediatric CF fecal samples reveals taxonomic dysbiosis that diminishes with age

**Figure 1:**
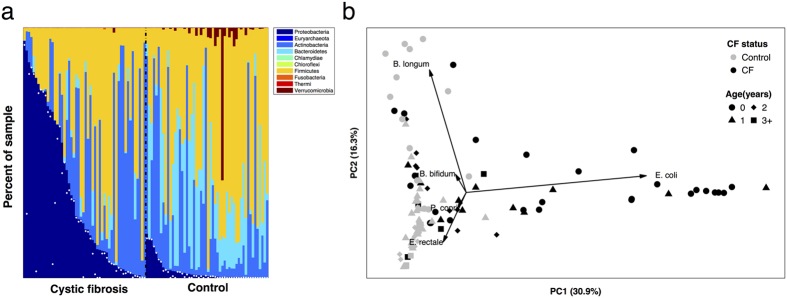
**(a)** The relative abundance of bacterial phyla among fecal samples from children with CF (left) and without CF (right). Samples are ordered for ease of display in decreasing abundance of Proteobacteria; relative abundance of *E. coli* is marked in each sample by a white dot. **(b)** A principal components analysis (PCA) of the taxonomic profile of each sample. The percent of variation explained by each of the first two components is noted on the axes and the top five loadings (scaled for ease of display) are illustrated. Evidently, the microbiota of CF fecal samples differ the most from non-CF samples at earlier source subject ages, driven largely by relative abundance of *E. coli* and *Bifidobacterium longum*.

In a previous cross-sectional microbiota analysis, we showed that *E. coli* was markedly more abundant in fecal samples from young children with CF than in those without CF^3^. This prior study focused on this striking *E. coli* dysbiosis, but did not characterize in detail how these differences related to the age of the source subjects, how these differences impacted the rest of the microbiota, and how community-wide dysbiosis links to *E. coli* abundance. To address these questions, here we obtained Illumina shotgun sequence reads from an expanded sample set, including a total of 104 fecal specimens collected over a period averaging approximately 12 months from 26 children (14 with CF and 12 without CF), comprising 52 CF and 52 non-CF samples, and characterized their microbiota using metagenomic phylogenetic analysis (MetaPhlAn^6^). We found that CF samples had a high relative abundance of *Proteobacteria* (including but not limited to *E. coli*) and *Actinobacteria* and low abundance of *Firmicutes*, *Bacteroidetes* and *Verrucomicrobia* compared to samples from children without CF (Fig. 1a; see also Supplementary Fig. S1). For this sample set, CF fecal microbiota also exhibited significantly lower α-diversity (Shannon index) than did non-CF microbiota (p < 0.0063, two-sided t-test). Importantly, however, this difference was no longer significant after excluding *E. coli* (p = 0.094; Methods), suggesting that the expansion of *E. coli* in CF fecal communities is not accompanied by a significant difference in the microbial diversity of the rest of the microbiota.

Going beyond phylum-level, we further characterized the taxa that differed significantly in relative abundance between CF and non-CF samples (Supplementary Table S2; Methods). We found that although *Firmicutes* was relatively depleted as a phylum in CF samples (P < 0.01), one order of *Firmicutes*, *Lactobacillales*, was significantly enriched in CF samples (P < 0.001), including the known pathogens *E. faecalis* and *E. faecium*, which are known to frequently exhibit antibiotic resistance^16^,^17^. In addition, the *Firmicutes* genus *Veillonella* was significantly enriched in CF samples (P < 10^−5^), including the species *V. parvula*, which has been identified in the lungs of children with CF^18^. In contrast, the *Firmicutes* order *Clostridiales* (which includes many taxa that contribute to GI immune homeostasis and development^19^,^20^) was significantly depleted in CF samples (P < 10^−4^); one of the only two *Clostridiales* species that were enriched in CF samples was *C. difficile*, well-known for its pathogenic potential after infancy^21^. Importantly, since environmental factors such as antibiotic exposure and breastfeeding can impact an infant’s microbiome^22^,^23^,^24^,^25^, we confirmed that analyses yielded similar results when excluding all samples from children who were breastfed at the time of collection, or samples taken after antibiotic treatment up to 60 days, as well as when controlling for *E. coli* abundance (see Supplementary Tables S3–S5).

To further characterize how the phylogenetic differences observed above change with age, we performed a principal components analysis (PCA) of the obtained taxonomic profiles. As shown in the resulting PCA plot (Fig. 1b), microbiota from control samples were largely distinguished from those of CF samples by the first component, owing mostly to differences in *E. coli* abundance (evidenced by the PCA loadings). Notably, however, CF samples from younger children were more clearly separated from younger control samples, while samples from older children with CF seemed to converge towards samples from older controls. The second principal component, predominantly governed by the relative abundance of *Bifidobacterium spp*, further separated younger children from older children in both groups. Next, to specifically examine the role of *E. coli* in CF and non-CF microbiota composition over time, we performed a similar PCA analysis after excluding the abundance of *E. coli* from the microbiota of each sample (Fig. S2; see Methods). The resulting CF and non-CF samples were still largely separated on the PCA plot, mostly owing to the abundances of the Actinobacterium *Bifidobacterium bifidum* (which is generally higher in CF samples) and the Firmicute *Eubacterium rectale* (lower in CF samples). Indeed, a statistical analysis of the abundances of the various species in CF versus non-CF samples while controlling for *E. coli* abundance confirmed that *Eubacterium rectale* (as well as several other species) had significantly lower relative abundance in CF samples (Supplementary Table S3). Again, the magnitude of the differences between CF and non-CF samples tended to diminish with source subject age (Fig. 1 and Supplementary Fig. S2), indicating that the taxonomic dysbiosis was most marked in infancy and waned over time.

### Functional metagenomic analysis shows differences in metabolic capacity between CF and non-CF pediatric fecal microbiota

To investigate potential functional differences between CF and non-CF fecal microbiota, shotgun metagenomic reads were mapped to the KEGG database to estimate the abundance of each KEGG orthology group (KO) in each sample (Methods). In total, we identified 13,840 KOs across the entire sample set, with an average of 5,725 KOs per sample. We then summed the abundances of all KOs associated with each microbial pathway (or module) to obtain a comprehensive functional profile of each sample (Methods).

**Figure 2:**
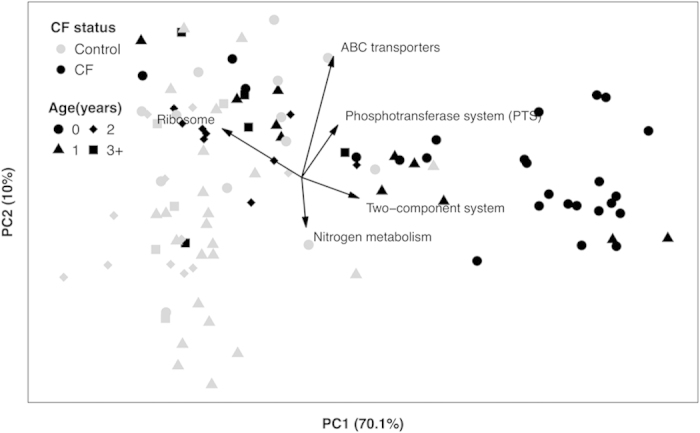
Principal component analysis (PCA) using pathway-level relative abundances of CF (black symbols) versus non-CF (gray symbols) samples. The percent of variation explained by each of the first two components is noted on the axes. The top five loadings (scaled for ease of display) are also illustrated.

A PCA of the resulting pathway-level abundance profiles demonstrated a clear distinction between samples from young children with CF (≤1 year) and those without CF (Fig. 2). Importantly, however, those distinctions were diminished in samples from older children, mirroring the pattern observed in the taxonomic profile (Fig. 1b). In addition, as observed for taxonomy, functional composition among samples from children with CF tended to differ between younger and older subjects much more than did those from children without CF. Using a novel normalization method that aims to correct potential biases that stem from using relative, rather than absolute, abundances (Methods) produced similar patterns and further highlighted the differences in functional capacity between CF and non-CF fecal microbiota (Fig. S3). These findings suggest that the GI microbiome in young children with CF have functional capacities that differ markedly from those without CF, but that this effect diminishes with age.

### Pediatric CF fecal microbiomes have altered capacities for fatty acid metabolism

We next used statistical analysis to identify significant differences in the relative abundance of specific bacterial functional pathways or modules that potentially underlie the separation observed in the PCA results above (Figs 2 and S3; Methods). We identified 17 pathways and 25 modules that were enriched in CF samples, and 36 pathways and 65 modules that were depleted in CF samples, relative to non-CF samples (p < 0.05, corrected for multiple comparisons; see Methods and Supplementary Tables S6–S9). Inspection of these functional differences revealed that multiple pathways and modules for fatty acid metabolism were differentially abundant in CF. Significantly, the KEGG fatty acid degradation pathway was enriched in CF, whereas the fatty acid biosynthesis pathway, as well as two fatty acid biosynthesis modules, were depleted in CF (Fig. S4 and Table 1). This decreased capacity of the CF fecal microbiota for fatty acid synthesis but an increased ability to metabolize fats overall might be expected if fatty acid availability was an important selective force for microbiota in the CF lumen. Examining the relative abundance of these pathways within subjects as a function of age again demonstrated that these differences in metabolic capacity between the CF and non-CF samples generally diminished with time (Fig. S4).

Because the CF fecal dysbiosis we observed previously^3^ was characterized by a marked relative enrichment for the Proteobacterium *E. coli*, particularly among the samples taken at earlier ages, we additionally set out to examine the contribution of *E. coli* to the functional differences in fatty acid metabolism reported above. To this end, we considered only the 63 samples that had a relative abundance of *E. coli* of <5% (since a substantial number of healthy samples, 7 out of 52, had at least 5% *E. coli*) and again used comparative analysis to identify differentially abundant pathways. We found that, despite the decreased sample size of this analysis, both the enrichment for genes encoding fatty acid degradation and the depletion of those encoding fatty acid biosynthesis in CF samples remained significant (p < 0.02 and p < 0.002, respectively, FDR <5%), indicating that the marked shifts in the abundance of fatty acid degradation and biosynthetic genes in the CF metagenomes could not be attributed solely to the differential abundance of *E. coli*, but rather involved the wider microbiota. In addition, we confirmed that the depletion of the fatty acid biosynthesis pathway in CF samples is not solely driven by *E. coli* by using an alternative sequence-based alignment analysis and removing short reads originating from *E. coli* genomes (see Methods). As for our taxonomic analyses described above, we further confirmed that our findings are not affected by excluding samples collected after antibiotic treatment or during breastfeeding (Supplementary Table S10).

### Pediatric CF fecal microbiomes have increased capacities for breakdown of the anti-inflammatory small-chain fatty acids butyrate and propionate, which correlate with fecal measures of inflammation. 

**Figure 3:**
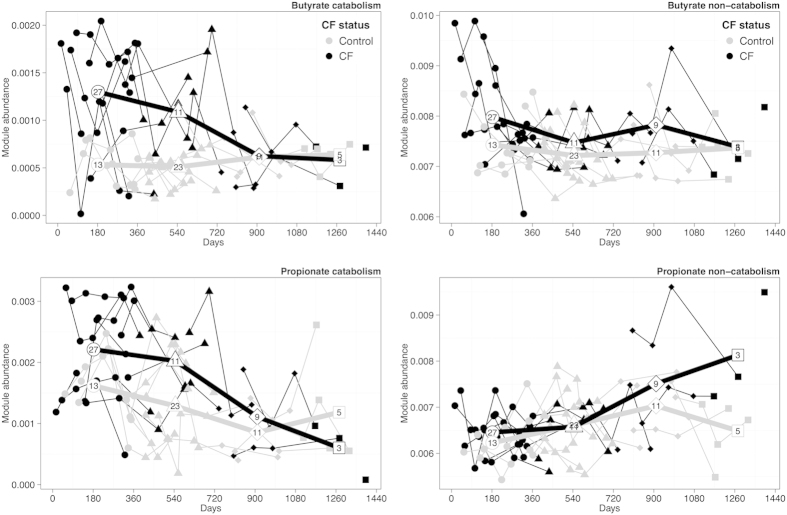
Temporal patterns in the abundance of butyrate and propionate modules in fecal samples from children with (black) vs. without (gray) CF. The abundance of each module is defined as the sum of the relative abundances of all the KOs associated with that module and is plotted by age of source subject. Lines connect samples from the same subject. The bold lines illustrate the average abundance of the module for all CF vs. all non-CF samples within a year of age (*i.e*., 0 to 365 days, 366 to 730 days, etc.), with each average plotted at the midpoint for each year. The number of samples available to calculate each average is shown inside the marker.

In addition to the above general shifts in fatty acid metabolism, we identified CF-associated enrichment specifically in the metabolism of butyrate and propionate – two short-chain fatty acids (SCFAs) produced and metabolized by GI microbiota and important for intestinal health^26^. In defining these pathways, however, KEGG does not distinguish between synthetic and degrading (or catabolic) processes. Considering the difference observed above between fatty acid biosynthesis and catabolism in relation to CF, we conducted a literature survey to manually partition the genes in each of these two SCFA pathways into two modules, one representing genes known to be associated with catabolism and the other representing genes encoding other (i.e., non-catabolic) enzymatic functions (Methods; see Supplementary Tables S11–S12 for full lists). In contrast to the trend observed above for fatty-acid metabolism, both the butyrate catabolism module and the butyrate non-catabolism module were enriched in CF samples; however, the CF enrichment level of the catabolism module was markedly more pronounced (p < 10^−6^ vs. p < 10^−3^ for catabolism and non-catabolism, respectively; Supplementary Table S13). Moreover, comparing the ratio between the average abundance of each of these modules in CF vs. non-CF samples, we detected a markedly more pronounced increase in the abundance of the catabolic module in CF vs. that of the non-catabolic module (1.95-fold vs. 1.07-fold for catabolism and non-catabolism, respectively). For propionate, only the catabolism module was enriched in CF, and again its relative abundance in CF vs. non-CF samples was much higher than that of the non-catabolic module (p < 10^−3^, 1.47-fold, vs. p = 0.56, 1.02-fold, for catabolism and non-catabolism, respectively; Supplementary Table S13). Plotting the relative abundance of these SCFA modules in CF and non-CF samples over time highlighted the more pronounced CF-associated enrichment of the catabolic modules (compared to the non-catabolic modules), again suggesting that these differences in SCFA metabolism diminish with age (Fig. 3).

The above results indicate that pediatric CF fecal microbiota have altered capacities for metabolism of fatty acids in general, and SCFAs in particular. Importantly, both butyrate and propionate are produced by the GI microbiota during fermentation of non-digestible starches and other carbohydrates^26^. In turn, both SCFAs (particularly butyrate) play important roles in GI epithelial health, including enterocyte nourishment and development, as well as ameliorating intestinal inflammation, reinforcing the epithelial defense barrier, and regulating intestinal motility, all of which are dysfunctional in humans and/or animals with CF mutations^2^. The observed enrichment of genes involved in catabolism of butyrate and propionate in the pediatric CF microbiota, which is likely to result in increased breakdown of these SCFAs, would be predicted to increase GI inflammation. In support of this prediction, we showed previously that measures of both fecal fat and inflammation in CF were highly correlated with the magnitude of CF-associated *E. coli* dysbiosis^3^. To directly explore the link between SCFA metabolism, fat content, and inflammation, we calculated the correlation between the overall abundance of genes for metabolism of butyrate and propionate, fecal fat content, and fecal calprotectin. We found a significant positive correlation between the fecal abundance of both the butyrate and propionate catabolism modules and fecal fat content (r = 0.61, p < 10^−4^ and r = 0.47, p < 10^−4^, respectively; Supplementary Table S14). We similarly found a significant positive correlation between the fecal abundance of these two modules and calprotectin (r = 0.5, p < 10^−4^ and r = 0.45, p < 10^−4^, respectively). Notably, the two corresponding non-catabolism modules were not significantly correlated with calprotectin (Supplementary Table S14). Combined, these findings could indicate that GI luminal fat selects for microbiota that, in turn, are pro-inflammatory, as schematized in the model in Supplementary Fig. S5.

## Discussion

Nutrient malabsorption, intestinal dysfunction, and malnutrition are among the most important and troubling early manifestations of CF. The malabsorption of fats in CF is largely due to inadequate secretion of the enzyme pancreatic lipase into the intestinal lumen, with contributions from other mechanisms^27^, resulting not only in fatty stools, but also to loss of nutritionally important dietary fat and fat-soluble vitamins. Our results suggest a model wherein excess dietary fat within the CF GI lumen also plays an indirect role in the intestinal inflammation that characterizes childhood CF GI disease by selecting for microbiota that preferentially degrade the SCFAs butyrate and propionate, important molecules for enteric health (Supplementary Fig. S5).

SCFAs are known to have multiple positive effects in the GI tract. For example, butyrate has both growth-promoting and anti-inflammatory effects on enteric epithelia^26^,^28^, and it was shown to ameliorate intestinal inflammation in animal models of colitis by promoting the differentiation of homeostatic regulatory T cells^20^. Many of its effects in the GI tract apparently are conveyed by inhibiting the activation of both NF-κB signaling and histone deacetylation^29^. Propionate, by contrast, is used as a substrate for gluconeogenesis and regulates cholesterol synthesis in the liver, potentially impacting nutritional status, but with less of a defined effect on inflammation^30^,^31^. SCFAs are produced by carbohydrate fermentation in the large intestine, largely by Firmicute bacteria of the order Clostridiales, including those in the genera *Eubacterium, Faecalibacterium, Ruminococcus*, and *Roseburia*^31^,^32^, many of which are depleted in human inflammatory bowel diseases^33^, and all of which were less abundant in the current study among CF microbiota (Supplementary Table S2). Moreover, SCFAs are important sources of energy salvage in people with malabsorption due to pancreatic insufficiency, a key manifestation of CF GI dysfunction^34^. While the enrichment for genes involved in butyrate and propionate catabolism relative to biosynthesis among CF metagenomes likely reflects an altered ratio of SCFA-producing versus SCFA-consuming taxa, it is challenging to determine exactly which species are responsible for the relative enrichment of catabolic genes in CF. Butyrate degradation, for example, is known to be associated with methanogenic archaea in the human colon^35^. An altered abundance of such archaea would have been reflected in our metagenomic (and thus gene content), but not taxonomic, analyses, as the taxonomic reference database had relatively little representation of archaea. Sulfate-reducing bacteria, including species of the genus *Desulfovibrio*, can also oxidize butyrate, and *Desulfovibrio* species are present in human feces^36^. Sulfate-reducing bacteria in the feces of diverse human populations have been shown to be capable of fermenting butyrate and propionate *ex vivo*^37^. Many of these sulfate-reducing bacteria have been shown to oxidize butyrate and other SCFAs using sulfate or nitrate as electron acceptors^12^,^38^,^39^; both sulfate and nitrate are present in the human GI tract^38^,^40^,^41^,^42^. The GI tracts of children with CF also contain elevated levels of the potential electron acceptor nitric oxide^43^. Therefore, the CF GI luminal environment would be predicted to be favorable for microbial butyrate catabolism, lending further support for our model. Furthermore, a previous metaproteomic study of CF fecal samples found evidence for a relative depletion of butyrate-producing bacteria, in support of our findings, but the species responsible were not identified^44^. The laboratory isolation and/or cultivation of most of these species is technically very challenging, and for some impossible, rendering further study of these concepts difficult.

Similarly, because butyrate and propionate are volatile acids, measurement of their abundances must be performed either on freshly collected fecal samples or on those that have been appropriately processed and stored in airtight containers^45^, neither of which was the case for our samples. In addition, 98% of SCFAs in the colon are absorbed rather than excreted^34^, and inflammatory and malabsorptive GI conditions are often associated with decreased colonic butyrate uptake and utilization^46^. Accordingly, a study comparing GI luminal SCFA measurements in children with vs. without CF would be required to test our model. Nevertheless, there is strong supportive evidence from animal models. For example, mice fed a high-fat diet had lower colonic abundances of butyrate-producing microbes, including *Roseburia*, and higher abundances of *Escherichia* and *Desulfovibrio*, than did mice on a normal diet, resulting in significant reductions in fecal butyrate and compromised GI host defenses that normalized with oral butyrate administration^47^.

While this study focused on bacteria, the GI tract microbiota clearly also includes both viruses and fungi, each of which could conceivably contribute to GI microbial community metabolism^48^,^49^. For example, the fungus *Aspergillus nidulans* has been shown to express a transporter for SCFAs (albeit with low affinity for either butyrate or propionate^50^). Therefore, future work will be required to define the contribution of non-bacterial taxa on community metabolism.

While people with CF frequently receive antibiotics to treat their respiratory disease^51^, and antibiotic treatment can at least transiently deplete butyrate-producing microbiota in the GI tract^52^, we showed previously that pediatric CF fecal dysbiosis was independent of recent antibiotic exposure within the prior 30 days^3^. In this study, we again confirmed that our findings were not likely impacted by antibiotic exposure by excluding all samples that were collected within 30 days of antibiotic treatment (15 samples; Supplementary Table S1), or even those collected within 60 days (20 samples), and repeating our analysis (see Supplementary Table S10). Nevertheless, antibiotics would be likely to contribute to functional depletion of butyrate production capacity by the microbiome. We also confirmed that restricting our analysis to a single sample from each individual did not markedly impact our findings (Supplementary Table S10). Finally, since breastfeeding is known to impact the composition of the GI microbiota^24^, we additionally confirmed that restricting our analysis to samples from non-breastfed infants did not significantly affected our results (Supplementary Table S10).

CF is caused by dysfunction of the epithelial transmembrane ion channel, the CF transmembrane regulator (CFTR); interestingly, butyrate has been shown to increase the expression of CFTR on the epithelial apical surface^53^,^54^. Therefore, should GI luminal butyrate abundance be decreased in children with CF, treatments that address this imbalance (such as therapies that modify the GI microbiota or replete luminal butyrate concentrations) could improve CF GI function and nutritional outcomes, and subsequent long-term health, through multiple mechanisms.

In conclusion, we found that the fecal microbiomes from children with CF exhibited taxonomic and functional differences from those of children without CF. Computational analysis of these microbiomes indicates that the pediatric CF GI microbiota are selected, at least in part, by the high abundance of unabsorbed luminal fatty acids, and that these CF microbiota are predicted to yield lower amounts of health-promoting SCFAs in the GI lumen (Supplementary Fig. S5). Future research will be required to verify the predicted depletion of butyrate and propionate in the GI tracts of children with CF, and to determine whether treatments to manipulate their microbiota lead to improved outcomes.

## Additional Information

**How to cite this article**: Manor, O. *et al*. Metagenomic evidence for taxonomic dysbiosis and functional imbalance in the gastrointestinal tracts of children with cystic fibrosis. *Sci. Rep*. **6**, 22493; doi: 10.1038/srep22493 (2016).

## Supplementary Material

Supplementary Information

## Figures and Tables

**Table 1:** Fatty acid metabolism functional differences between CF and non-CF fecal samples.

Functional assignment^a^	#non-zero KOs^b^	Shift^c^	P value^d^
ko00650: Butyrate metabolism	60	E	3.83e–07
ko00640: Propionate metabolism	53	E	1.01e–06
ko00071: Fatty acid degradation	28	E	5.70e–04
ko00061: Fatty acid biosynthesis	22	D	2.43e–08
M00083: Fatty acid biosynthesis, elongation	12	D	5.34e–10
M00082: Fatty acid biosynthesis, initiation	11	D	2.06e–05

^a^Numbers beginning with ‘ko’ indicate a functional pathway; those beginning with an ‘M’ indicate a functional module.

^b^See Methods.

^c^Enriched (E) or depleted (D) in CF.

^d^Wilcoxon rank-sum test for CF vs. non-CF.
