# Supplementary material for: Metagenomic evidence for taxonomic dysbiosis and functional imbalance in the gastrointestinal tracts of children with cystic fibrosis
Source: Sci Rep. 2016 Mar 4;6:22493. doi: 10.1038/srep22493 (PMC4778032; doi:10.1038/srep22493)
Supplement: Supplementary Information [file srep22493-s1.pdf]

Supplementary Information for: Metagenomic evidence for taxonomic dysbiosis and functional imbalance in the gastrointestinal tracts of children with cystic fibrosis

Ohad Manor [1,*], Roie Levy [1,*], Christopher E. Pope [2], Hillary S. Hayden [3], Mitchell J. Brittnacher [3], Rogan Carr [1], Matthew C. Radey [3], Kyle R. Hager [3], Sonya L. Heltshe [2,6], Bonnie W. Ramsey [2,6], Samuel I. Miller [1,3,4], Lucas R. Hoffman [2,3,6,†], Elhanan Borenstein [1,5,7,†]

Departments of [1]Genome Sciences, [2]Pediatrics, [3]Microbiology, [4]Medicine, and [5]Computer Science and Engineering, University of Washington, Seattle; [6]Seattle Children's Hospital, Washington; [7]Santa Fe Institute, New Mexico

* These authors contributed equally to this work
† Co-corresponding authors (LRH: lhoffm@uw.edu, EB: elbo@uw.edu)

**Figure/Table**	**Description**
Figure S1	The relative abundance of bacterial phyla among fecal samples separated by subject.
Figure S2	Principal components analysis (PCA) of the taxonomic profiles after excluding *E. coli* or samples that had antibiotic exposure.
Figure S3	Principal component analysis (PCA) of the functional profiles using pathway-level abundances.
Figure S4	Temporal patterns in the abundance of the fatty acid degradation and biosynthesis pathways.
Figure S5	A model for how CF GI dysfunction and dysbiosis can lead to inflammation.
Table S1	Sample information
Table S2	Differentially abundant species in CF vs. non-CF samples
Table S3	Differentially abundant species when controlling for E. Coli
Table S4	Differentially abundant species when controlling for antibiotic exposure
Table S5	Differentially abundant species when controlling for breastfeeding
Table S6	KEGG Pathways found to be enriched in CF samples
Table S7	KEGG Pathways found to be depleted in CF samples
Table S8	KEGG Modules found to be enriched in CF samples
Table S9	KEGG Modules found to be depleted in CF samples
Table S10	Results for subsets of samples excluding antibiotics-treated or breastfed children
Table S11	List of KOs in the butyrate catabolism module
Table S12	List of KOs in the propionate catabolism module
Table S13	Butyrate and propionate enrichment statistics in CF samples
Table S14	Butyrate and propionate correlations with inflammation and fat content

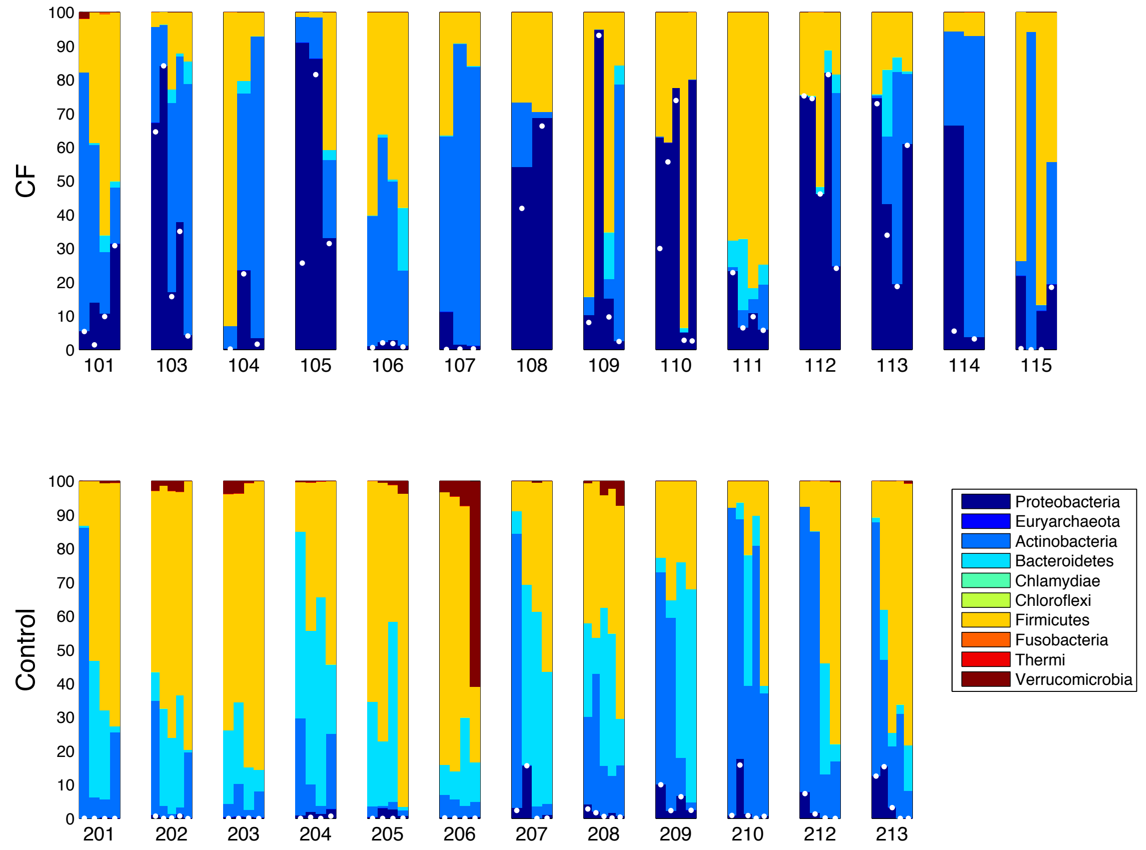

Figure S1: The relative abundance of bacterial phyla among fecal samples from children with CF (top) and without CF (bottom), separated by subject. Samples are ordered by age. Relative abundance of *E. coli* is marked in each sample by a white dot.

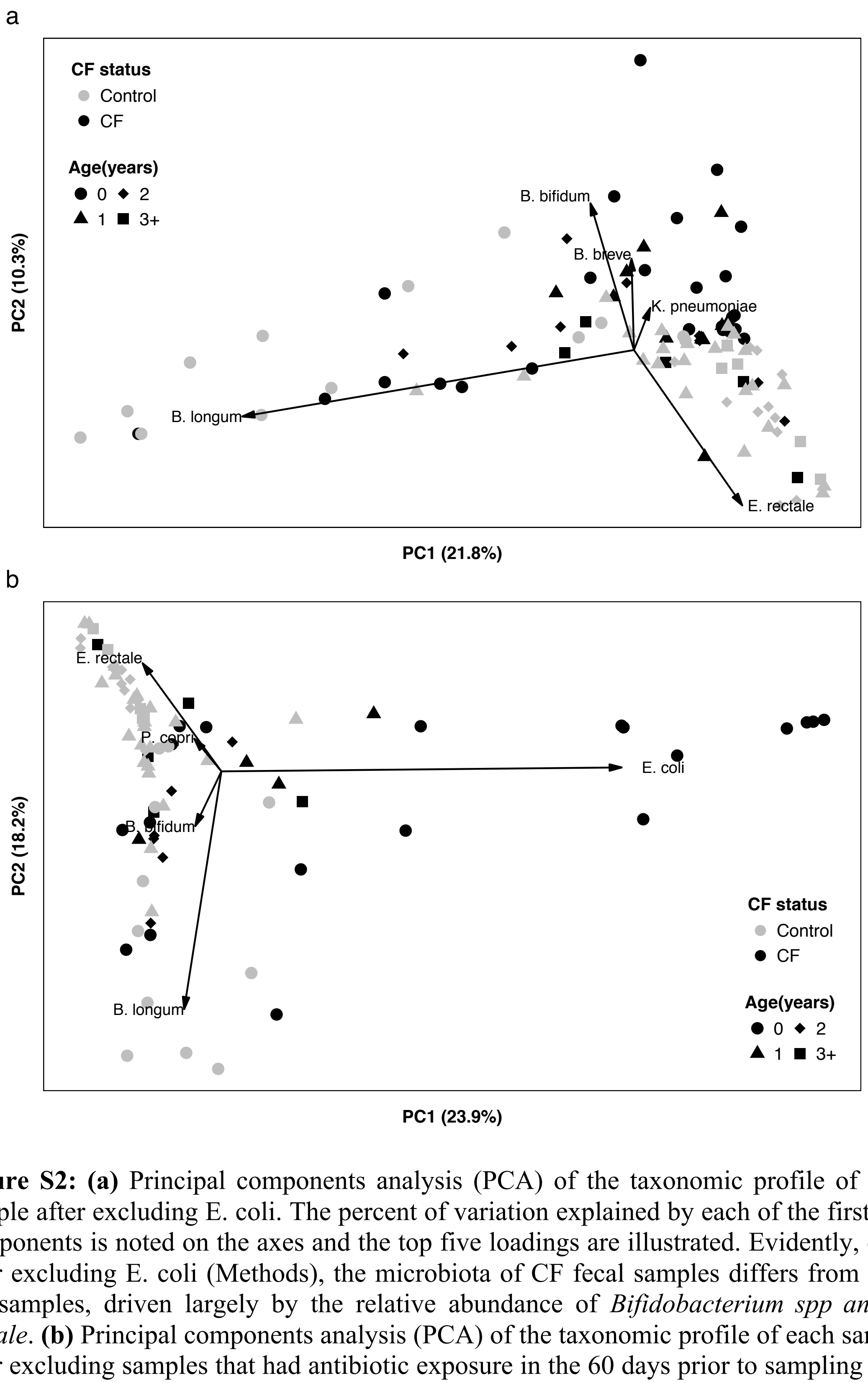

Figure S2: (a) Principal components analysis (PCA) of the taxonomic profile of each sample after excluding E. coli. The percent of variation explained by each of the first two components is noted on the axes and the top five loadings are illustrated. Evidently, even after excluding E. coli (Methods), the microbiota of CF fecal samples differs from non-CF samples, driven largely by the relative abundance of *Bifidobacterium spp and E. rectale*. **(b)** Principal components analysis (PCA) of the taxonomic profile of each sample after excluding samples that had antibiotic exposure in the 60 days prior to sampling (and compare to Figure 1b). The percent of variation explained by each of the first two components is noted on the axes and the top five loadings are illustrated.

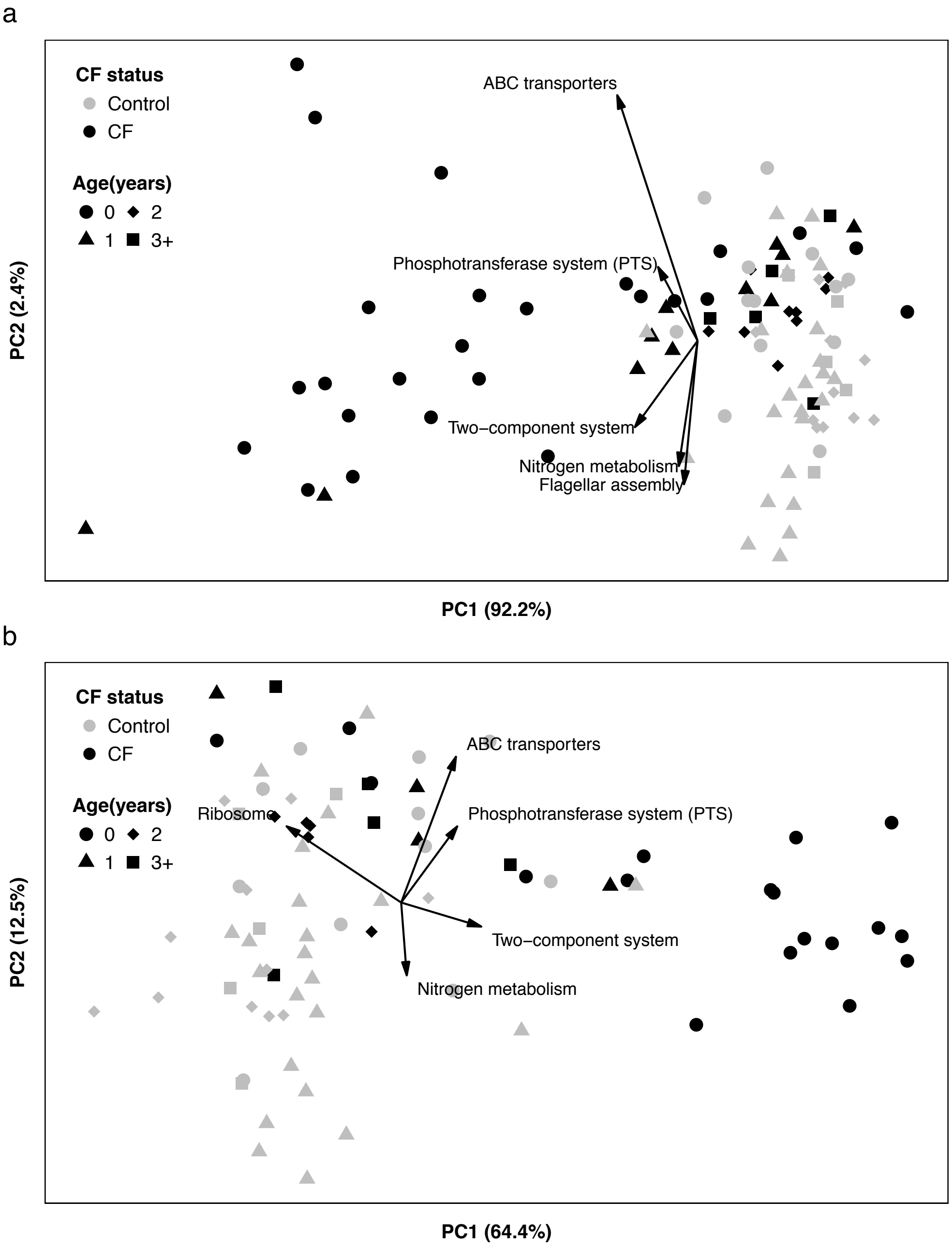

Figure S3: (a) Principal component analysis (PCA) using pathway-level abundances corrected by MUSiCC (Methods). Applying MUSiCC to the metagenomic functional profile resulted in better separation between CF (black symbols) and non-CF (gray symbols) samples. The percent of variation explained by each of the first two components is noted on the axes. The top five loadings are also illustrated. **(b)** Principal components analysis (PCA) using pathway-level abundances after excluding samples that had antibiotic exposure in the 60 days prior to sampling (and compare to Figure 2). The percent of variation explained by each of the first two components is noted on the axes and the top five loadings are illustrated.

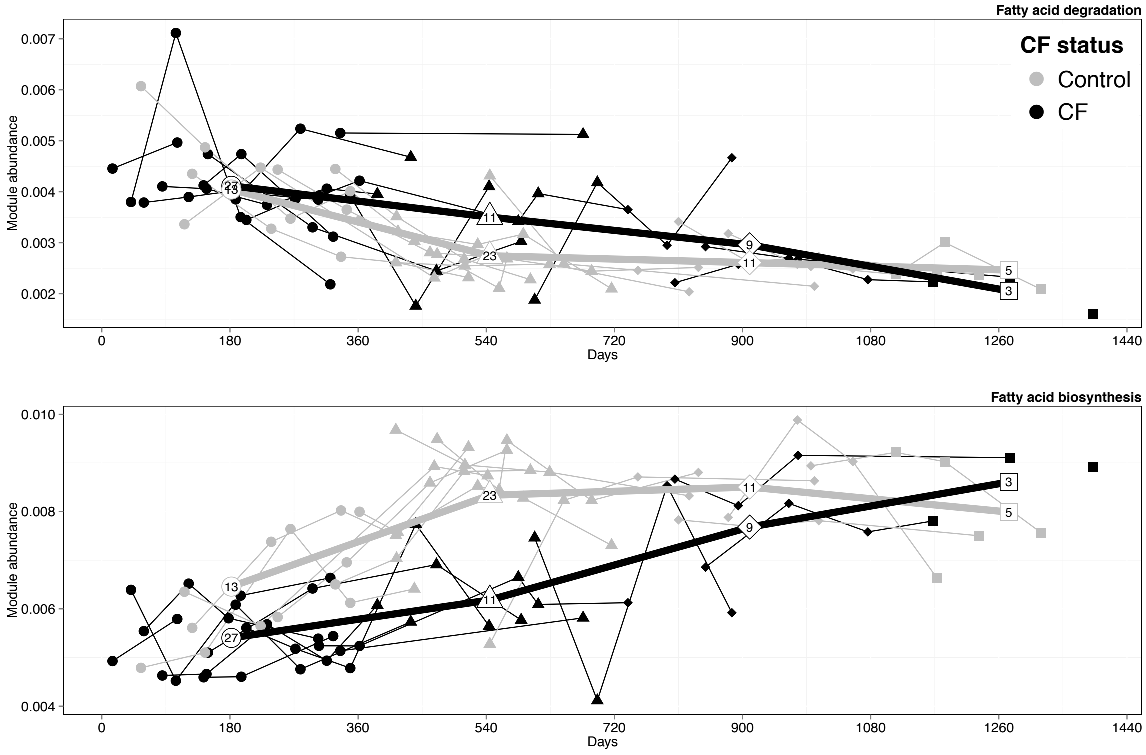

Figure S4: Temporal patterns in the abundance of the fatty acid degradation and biosynthesis pathways in fecal samples from children with (black) vs. without (gray) CF. Lines connect samples from the same subject. The bold lines illustrate the average abundance of these pathways for all CF vs. all non-CF samples within a year of age, as in Figure 3. The number of samples available to calculate each average is shown inside the marker.

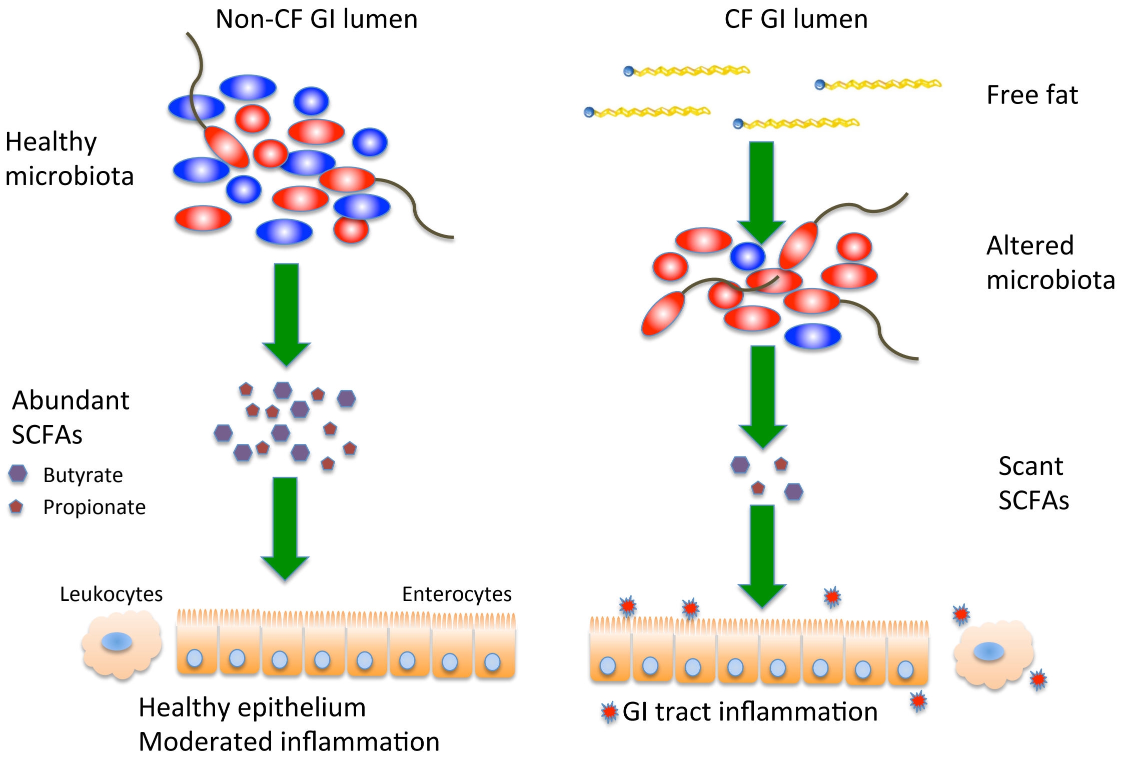

Figure S5: A model for how CF GI dysfunction and dysbiosis can lead to inflammation. In the healthy infant GI lumen (left), normal GI microbiota metabolize resistant starches and other polysaccharides to produce short-chain fatty acids (SCFAs), including butyrate and propionate, which in turn regulate GI epithelial health and moderate inflammatory responses through interactions with the GI epithelium and regulatory leukocytes. In the CF GI tract (right), according to the model, unabsorbed luminal fats (free fatty acids and phospholipids) would select for altered GI microbiota that, as a community, produce much lower amounts of the SCFAs butyrate and propionate, resulting in higher levels of GI inflammation.

Table S1. Subject and clinical information for all samples included in the study

Subject information						Specimen information		Diet information*			Antibiotics usage**		Sequencing information	
sample	rep	Used in [3]	date	age (days)	CF	fat	Calprotectin (ug/g)	Breastfed	Formula	Food	abx30	abx60	reads	reads after human filtering
101	1	Yes	4/13/2011	149	1	1.00	120.33	1	1	1	0	0	47,972,168	47,960,612
101	2	Yes	9/7/2011	296	1	1.00	64.96	1	1	1	0	0	43,951,804	43,929,762
101	3	Yes	2/28/2012	470	1	0.00	61.52	0	1	1	1	1	16,340,540	16,327,136
101	4	Yes	6/26/2012	589	1	20.00	88.92	0	0	1	0	0	30,788,022	30,783,284
103	1	Yes	5/17/2011	305	1	45.45	422.82	0	1	1	0	0	30,174,456	30,157,232
103	2	Yes	7/13/2011	362	1	33.33	378.32	0	1	1	0	1	228,350,288	228,162,744
103	3	Yes	2/21/2012	585	1	20.00	362.87	0	1	1	0	0	21,546,481	21,481,038
103	4	Yes	3/20/2012	613	1	40.00	96.96	0	0	1	1	1	29,934,965	29,890,476
103	5	No	7/24/2012	739	1	30.77	N/A	0	0	1	0	0	28,051,824	28,023,842
104	1	No	4/10/2011	1392	1	N/A	N/A	0	0	1	0	0	19,488,286	19,483,532
104	2	No	2/6/2012	1694	1	46.15	N/A	0	0	1	0	0	118,374,128	88,947,686
104	3	No	8/15/2012	1885	1	7.69	N/A	0	0	1	0	0	21,547,558	21,534,636
105	1	Yes	6/14/2011	143	1	64.29	393.39	0	1	1	1	1	26,925,888	26,923,849
105	2	Yes	8/6/2011	196	1	40.00	180.20	0	1	1	1	1	112,071,808	111,991,740
105	3	Yes	12/13/2011	325	1	33.33	242.89	0	1	1	0	1	18,240,332	17,860,674
106	1	Yes	5/11/2011	805	1	4.76	53.11	0	0	1	0	0	23,965,586	23,959,434
106	2	Yes	8/8/2011	894	1	41.67	14.63	0	0	1	0	0	13,798,185	13,788,591
106	3	Yes	10/31/2011	978	1	30.00	23.09	0	0	1	0	0	147,891,358	147,326,804
106	4	No	8/23/2012	1275	1	15.38	N/A	0	0	1	0	0	114,902,670	113,603,090
107	1	Yes	5/16/2011	188	1	N/A	N/A	1	1	1	0	0	29,699,935	29,309,453
107	2	Yes	8/15/2011	279	1	14.29	153.02	0	1	1	0	0	26,521,755	26,498,960
107	3	Yes	1/17/2012	434	1	11.11	69.72	0	0	1	0	0	16,598,024	14,828,649
108	2	Yes	6/23/2011	335	1	0.00	39.87	0	1	1	1	1	15,037,145	15,036,004
108	4	Yes	5/29/2012	676	1	8.33	65.53	0	0	1	1	1	11,744,104	11,743,083
109	2	Yes	8/12/2011	608	1	18.18	404.87	0	0	1	0	0	34,721,083	34,701,114
109	3	Yes	11/8/2011	696	1	30.00	80.21	0	0	1	1	1	13,169,942	13,159,697
109	4	Yes	2/14/2012	794	1	15.38	242.79	0	0	1	0	0	150,851,468	149,910,156
109	5	No	5/15/2012	885	1	21.43	N/A	0	0	1	0	0	65,128,312	65,108,594
110	1	Yes	6/1/2011	203	1	66.67	395.19	1	1	1	0	0	41,139,696	35,754,702
110	2	Yes	8/9/2011	272	1	28.57	239.71	1	1	1	0	0	46,126,719	46,110,725
110	3	Yes	10/25/2011	349	1	20.00	80.11	1	1	1	1	1	27,603,964	27,601,446
110	4	Yes	1/25/2012	441	1	30.77	171.03	0	0	1	0	1	42,963,327	42,923,371
110	5	Yes	5/7/2012	544	1	0.00	115.33	0	0	1	1	1	85,354,338	85,275,932
111	1	Yes	8/12/2011	848	1	13.04	N/A	0	0	1	1	1	96,019,756	55,152,696
111	2	Yes	12/7/2011	965	1	25.00	73.15	0	0	1	1	1	17,879,913	17,867,848
111	3	Yes	3/27/2012	1076	1	20.00	227.75	0	0	1	1	1	10,515,511	10,512,505
111	4	No	6/26/2012	1167	1	14.29	N/A	0	0	1	0	0	60,834,654	60,809,238
112	1	Yes	5/31/2011	85	1	66.67	395.73	1	0	0	0	0	18,034,920	18,033,068
112	2	Yes	8/1/2011	147	1	77.78	330.22	1	0	0	0	0	18,501,677	18,494,717
112	3	Yes	10/25/2011	232	1	45.45	113.59	1	0	1	0	0	114,788,076	114,561,160
112	4	Yes	1/17/2012	316	1	60.00	415.29	1	0	1	0	0	38,108,741	38,097,697
112	5	Yes	3/28/2012	387	1	50.00	170.40	1	0	1	0	1	17,106,886	17,104,100
113	1	Yes	6/14/2011	59	1	N/A	324.88	1	0	0	0	0	17,067,857	17,063,976
113	2	Yes	8/16/2011	122	1	60.00	61.10	1	1	0	0	0	87,981,958	87,772,716
113	3	Yes	10/11/2011	178	1	57.14	51.07	1	1	1	0	0	29,664,904	29,626,632
113	4	Yes	2/14/2012	304	1	0.00	48.20	1	1	1	0	0	242,331,984	242,134,728
114	1	Yes	8/2/2011	15	1	N/A	N/A	1	0	0	0	0	69,769,882	69,166,498
114	2	Yes	11/1/2011	106	1	N/A	N/A	1	0	0	0	0	114,760,448	114,719,328
115	1	No	10/18/2011	41	1	N/A	N/A	1	0	0	1	1	102,510,112	102,488,384
115	2	No	12/20/2011	104	1	N/A	N/A	1	0	0	0	0	73,980,570	53,197,486
115	3	No	3/20/2012	195	1	44.44	64.55	1	1	1	0	0	39,909,174	39,892,996
115	4	No	7/24/2012	321	1	11.11	N/A	0	1	1	0	0	60,383,956	60,380,512
201	1	Yes	12/13/2011	545	0	1.00	17.86	1	0	1	0	0	75,920,684	75,883,224
201	2	Yes	3/26/2012	649	0	1.00	29.41	0	0	0	0	0	103,040,640	102,916,028
201	3	Yes	7/8/2012	753	0	0.00	14.11	0	0	0	0	0	27,175,030	27,172,536
201	4	No	3/13/2013	1001	0	7.69	N/A	0	0	1	0	0	87,692,998	87,683,950
202	1	Yes	12/11/2011	471	0	0.00	14.83	0	0	1	0	0	154,061,672	154,002,478
202	2	Yes	3/18/2012	569	0	1.00	19.57	0	0	1	0	0	123,205,128	123,059,836
202	3	Yes	7/15/2012	688	0	0.00	11.53	0	0	1	0	0	29,249,752	29,242,200
202	4	Yes	12/12/2012	838	0	1.00	34.59	0	0	1	0	0	48,971,798	48,955,898
202	5	No	3/7/2012	558	0	0.00	N/A	0	0	1	0	0	28,181,886	28,179,714
203	1	Yes	2/1/2012	413	0	0.00	3.92	0	0	1	0	0	139,037,952	138,934,212
203	2	Yes	5/8/2012	510	0	1.00	11.07	0	0	1	0	0	175,816,548	175,628,576
203	3	Yes	9/4/2012	629	0	0.00	2.87	0	0	1	0	0	147,204,014	147,157,186
203	4	No	3/19/2013	825	0	1.00	N/A	0	0	1	0	0	86,868,482	86,854,782
204	1	Yes	1/22/2012	810	0	0.00	34.77	0	0	1	0	0	136,015,330	135,924,794
204	2	Yes	4/29/2012	908	0	11.11	24.68	0	0	1	0	0	126,288,088	126,135,088
204	3	Yes	8/6/2012	1007	0	0.00	19.31	0	0	1	0	0	39,590,926	39,581,330
204	4	No	3/19/2013	1232	0	0.00	N/A	0	0	1	0	0	61,058,054	61,045,170
205	1	Yes	1/30/2012	996	0	1.00	55.59	0	0	1	0	0	98,292,760	98,265,004
205	2	Yes	5/28/2012	1115	0	1.00	10.18	0	0	1	0	0	71,778,042	71,723,004
205	3	Yes	8/5/2012	1184	0	14.29	4.11	0	0	1	0	0	26,420,496	26,415,106
205	4	No	12/18/2012	1319	0	11.11	N/A	0	0	1	0	0	27,650,298	27,647,086
206	1	Yes	1/30/2012	880	0	1.00	11.31	0	0	1	0	0	134,607,950	132,854,534
206	2	Yes	5/6/2012	977	0	9.09	63.48	0	0	1	0	0	50,422,842	50,385,980
206	3	No	7/23/2012	1055	0	1.00	N/A	0	0	1	0	0	64,248,092	64,239,986
206	4	Yes	11/18/2012	1173	0	6.67	72.20	0	0	1	0	0	58,744,554	58,721,726
207	1	Yes	2/13/2012	328	0	0.00	49.46	1	0	1	0	0	86,402,812	86,342,204
207	2	Yes	5/9/2012	414	0	1.00	9.96	0	0	1	0	0	127,378,112	127,270,034
207	3	Yes	8/12/2012	509	0	0.00	9.39	0	0	1	0	0	91,030,482	91,003,724
207	4	Yes	11/13/2012	602	0	0.00	87.51	0	0	1	0	0	112,277,738	112,222,140
208	1	Yes	3/28/2012	362	0	1.00	30.51	1	0	1	0	0	95,164,320	95,022,504
208	2	Yes	5/21/2012	416	0	1.00	8.97	0	0	1	0	0	66,011,034	65,993,792

208	3	Yes	9/10/2012	528	0	1.00	-1.34	0	0	1	0	0	62,416,560	62,397,566
208	4	Yes	11/13/2012	592	0	0.00	35.48	0	0	1	0	0	72,853,264	72,840,674
208	5	No	3/17/2013	716	0	0.00	N/A	0	0	1	0	0	99,237,988	99,219,860
209	1	Yes	2/23/2012	247	0	0.00	22.62	1	0	1	0	0	136,394,272	136,370,124
209	2	Yes	5/30/2012	344	0	1.00	27.58	0	0	1	0	0	48,734,108	48,723,324
209	3	Yes	9/24/2012	461	0	0.00	2.06	0	0	1	0	0	77,317,094	77,108,742
209	4	Yes	1/10/2013	569	0	0.00	12.94	0	0	1	0	0	155,357,400	155,118,968
210	1	Yes	2/28/2012	55	0	16.67	281.97	0	1	0	0	0	139,542,344	139,528,886
210	2	Yes	5/28/2012	145	0	N/A	N/A	0	1	1	0	0	51,580,806	51,572,940
210	3	Yes	9/25/2012	265	0	0.00	55.05	0	1	1	0	0	63,861,708	63,847,612
210	4	No	12/18/2012	349	0	0.00	N/A	0	1	1	1	1	23,410,466	23,408,724
210	5	No	3/18/2013	439	0	1.00	N/A	0	0	1	1	1	116,894,722	116,874,958
212	1	Yes	8/14/2012	223	0	0.00	5.94	1	1	0	0	1	17,100,150	17,089,478
212	2	Yes	4/29/2012	116	0	0.00	26.65	1	0	0	0	0	61,242,420	61,206,736
212	4	No	6/29/2013	542	0	N/A	N/A	1	0	1	0	0	91,382,566	91,369,180
212	5	No	4/15/2013	467	0	N/A	N/A	1	0	1	0	0	33,700,722	33,692,388
213	1	Yes	6/4/2012	127	0	11.11	35.43	0	1	0	0	0	74,109,540	74,092,616
213	2	Yes	9/23/2012	238	0	1.00	21.78	0	1	1	0	0	83,985,518	83,944,962
213	3	Yes	12/30/2012	336	0	0.00	67.43	0	0	1	0	0	77,911,810	77,890,722
213	4	No	3/17/2013	413	0	0.00	N/A	0	0	1	0	0	44,597,980	44,593,808
213	5	No	6/27/2013	515	0	N/A	N/A	0	0	1	0	0	90,800,782	90,787,166

* 0 indicates no such exposure, 1 indicates exposure ** Exposure to antibiotics within 30 days and 60 days prior to collection, respectively

Table S2. Species found to be differentially abundant in CF or control samples

Phylum	Class	Order	Family	Genus	Species	p-value*	Abundant in**
Actinobacteria	Actinobacteria	Actinomycetales	Actinomycetaceae	Actinomyces	odontolyticus	0.0072532	CF
Actinobacteria	Actinobacteria	Actinomycetales	Micrococcaceae	Rothia	mucilaginosa	0.01450747	CF
Firmicutes	Bacilli	Lactobacillales	Enterococcaceae	Enterococcus	faecalis	0.00013375	CF
Firmicutes	Bacilli	Lactobacillales	Enterococcaceae	Enterococcus	faecium	0.00182504	CF
Firmicutes	Bacilli	Lactobacillales	Streptococcaceae	Streptococcus	mitis	0.01022964	CF
Firmicutes	Bacilli	Lactobacillales	Streptococcaceae	Streptococcus	parasanguinis	0.00010819	CF
Firmicutes	Clostridia	Clostridiales	Clostridiaceae	Clostridium	difficile	0.01301154	CF
Firmicutes	Clostridia	Clostridiales	Clostridiaceae	Clostridium	perfringens	1.7849E-07	CF
Firmicutes	Clostridia	Clostridiales	ales_Family_XI_Incert	Anaerococcus	unclassified	5.17E-04	CF
Firmicutes	Clostridia	Clostridiales	ales_Family_XI_Incert	Finegoldia	magna	1.83E-03	CF
Firmicutes	Negativicutes	Selenomonadales	Veillonellaceae	Megasphaera	micronuciformis	0.00012521	CF
Firmicutes	Negativicutes	Selenomonadales	Veillonellaceae	Veillonella	atypica	5.9523E-06	CF
Firmicutes	Negativicutes	Selenomonadales	Veillonellaceae	Veillonella	dispar	6.9323E-09	CF
Firmicutes	Negativicutes	Selenomonadales	Veillonellaceae	Veillonella	parvula	0.00015861	CF
Firmicutes	Negativicutes	Selenomonadales	Veillonellaceae	Veillonella	unclassified	0.0195996	CF
Proteobacteria	Gammaproteobacteria	Enterobacteriales	Enterobacteriaceae	Citrobacter	unclassified	0.00205917	CF
Proteobacteria	Gammaproteobacteria	Enterobacteriales	Enterobacteriaceae	Enterobacter	cloacae	0.00351119	CF
Proteobacteria	Gammaproteobacteria	Enterobacteriales	Enterobacteriaceae	Enterobacter	unclassified	0.00939806	CF
Proteobacteria	Gammaproteobacteria	Enterobacteriales	Enterobacteriaceae	Escherichia	coli	6.4355E-10	CF
Proteobacteria	Gammaproteobacteria	Enterobacteriales	Enterobacteriaceae	Klebsiella	pneumoniae	5.1808E-06	CF
Proteobacteria	Gammaproteobacteria	Enterobacteriales	Enterobacteriaceae	Klebsiella	unclassified	0.01615305	CF
Actinobacteria	Actinobacteria	Coriobacteriales	Coriobacteriaceae	Eggerthella	lenta	0.00016069	Control
Actinobacteria	Actinobacteria	Coriobacteriales	Coriobacteriaceae	Gordonibacter	pamelaeae	1.2765E-06	Control
Bacteroidetes	Bacteroidia	Bacteroidales	Bacteroidaceae	Bacteroides	caccae	3.8676E-05	Control
Bacteroidetes	Bacteroidia	Bacteroidales	Bacteroidaceae	Bacteroides	intestinalis	0.00194953	Control
Bacteroidetes	Bacteroidia	Bacteroidales	Bacteroidaceae	Bacteroides	ovatus	7.4882E-06	Control
Bacteroidetes	Bacteroidia	Bacteroidales	Bacteroidaceae	Bacteroides	unclassified	2.1833E-05	Control
Bacteroidetes	Bacteroidia	Bacteroidales	Bacteroidaceae	Bacteroides	vulgatus	6.1512E-07	Control
Bacteroidetes	Bacteroidia	Bacteroidales	Bacteroidaceae	Bacteroides	xylanisolvens	2.2474E-05	Control
Bacteroidetes	Bacteroidia	Bacteroidales	Rikenellaceae	Alistipes	putredinis	0.00021083	Control
Bacteroidetes	Bacteroidia	Bacteroidales	Rikenellaceae	Alistipes	shahii	0.00036713	Control
Firmicutes	Clostridia	Clostridiales	Clostridiaceae	Clostridium	asparagiforme	8.6113E-05	Control
Firmicutes	Clostridia	Clostridiales	Clostridiaceae	Clostridium	bartlettii	4.5371E-07	Control
Firmicutes	Clostridia	Clostridiales	Clostridiaceae	Clostridium	bolteae	0.00115164	Control
Firmicutes	Clostridia	Clostridiales	Clostridiaceae	Clostridium	cf	0.00034936	Control
Firmicutes	Clostridia	Clostridiales	Clostridiaceae	Clostridium	hathewayi	9.6942E-10	Control
Firmicutes	Clostridia	Clostridiales	Clostridiaceae	Clostridium	leptum	0.00261205	Control
Firmicutes	Clostridia	Clostridiales	Clostridiaceae	Clostridium	scindens	0.0213679	Control
Firmicutes	Clostridia	Clostridiales	Clostridiaceae	Clostridium	symbiosum	0.00201465	Control
Firmicutes	Clostridia	Clostridiales	Clostridiaceae	Pseudoflavonifractor	capillosus	5.16E-11	Control
Firmicutes	Clostridia	Clostridiales	Clostridiales_uncl	Blautia	unclassified	1.08E-02	Control
Firmicutes	Clostridia	Clostridiales	Eubacteriaceae	Eubacterium	eligens	5.8761E-05	Control
Firmicutes	Clostridia	Clostridiales	Eubacteriaceae	Eubacterium	rectale	0.00120544	Control
Firmicutes	Clostridia	Clostridiales	Eubacteriaceae	Eubacterium	siraeum	0.00312883	Control
Firmicutes	Clostridia	Clostridiales	Eubacteriaceae	Eubacterium	ventriosum	0.00985228	Control
Firmicutes	Clostridia	Clostridiales	Lachnospiraceae	Anaerostipes	caccae	0.00030074	Control
Firmicutes	Clostridia	Clostridiales	Lachnospiraceae	Butyrivibrio	unclassified	6.5593E-05	Control
Firmicutes	Clostridia	Clostridiales	Lachnospiraceae	Roseburia	intestinalis	1.6347E-07	Control
Firmicutes	Clostridia	Clostridiales	Lachnospiraceae	Roseburia	inulinivorans	0.00042047	Control
Firmicutes	Clostridia	Clostridiales	Ruminococcaceae	Anaerotruncus	colihominis	3.3355E-07	Control
Firmicutes	Clostridia	Clostridiales	Ruminococcaceae	Faecalibacterium	cf	0.00067356	Control
Firmicutes	Clostridia	Clostridiales	Ruminococcaceae	Faecalibacterium	prausnitzii	4.8396E-05	Control
Firmicutes	Clostridia	Clostridiales	Ruminococcaceae	Faecalibacterium	unclassified	0.00029699	Control
Firmicutes	Clostridia	Clostridiales	Ruminococcaceae	Ruminococcus	albus	0.01144006	Control
Firmicutes	Clostridia	Clostridiales	Ruminococcaceae	Ruminococcus	bromii	7.2634E-06	Control
Firmicutes	Clostridia	Clostridiales	Ruminococcaceae	Ruminococcus	lactaris	0.01424808	Control
Firmicutes	Clostridia	Clostridiales	Ruminococcaceae	Ruminococcus	obeum	0.01386666	Control
Firmicutes	Clostridia	Clostridiales	Ruminococcaceae	Subdoligranulum	variabile	0.00019518	Control
Firmicutes	Erysipelotrichi	Erysipelotrichales	Erysipelotrichaceae	Coprobacillus	bacterium	0.00027888	Control
Firmicutes	Erysipelotrichi	Erysipelotrichales	Erysipelotrichaceae	Holdemania	filiformis	0.00234579	Control
Firmicutes	Negativicutes	Selenomonadales	Veillonellaceae	Dialister	invisus	0.00064223	Control
Proteobacteria	Deltaproteobacteria	Desulfovibrionales	Desulfovibrionaceae	Bilophila	wadsworthia	0.01876439	Control
Proteobacteria	Deltaproteobacteria	Desulfovibrionales	Desulfovibrionaceae	Desulfovibrio	desulfuricans	6.0021E-08	Control
Verrucomicrobia	Verrucomicrobiae	Verrucomicrobiales	Verrucomicrobiaceae	Akkermansia	muciniphila	1.69E-07	Control

* Wilcoxon rank-sum test p-value when comparing between CF and control samples; **The sample set in which the species is more abundant.

Table S3. Species found to be differentially abundant in CF or control samples, when controlling for the abundance of E. Coli.

Phylum	Class	Order	Family	Genus	Species	p-value*	Abundant in**
Actinobacteria	Actinobacteria	Actinomycetales	Actinomycetaceae	Actinomyces	odontolyticus	0.00107511	CF
Actinobacteria	Actinobacteria	Actinomycetales	Micrococcaceae	Rothia	mucilaginosa	0.00255669	CF
Firmicutes	Bacilli	Lactobacillales	Enterococcaceae	Enterococcus	faecalis	9.9848E-05	CF
Firmicutes	Bacilli	Lactobacillales	Enterococcaceae	Enterococcus	faecium	0.00149409	CF
Firmicutes	Bacilli	Lactobacillales	Streptococcaceae	Streptococcus	mitis	0.00135023	CF
Firmicutes	Bacilli	Lactobacillales	Streptococcaceae	Streptococcus	parasanguinis	3.7353E-06	CF
Firmicutes	Bacilli	Lactobacillales	Streptococcaceae	Streptococcus	salivarius	0.0038952	CF
Firmicutes	Bacilli	Lactobacillales	Streptococcaceae	Streptococcus	vestibularis	0.01558716	CF
Firmicutes	Clostridia	Clostridiales	Clostridiaceae	Clostridium	difficile	0.00948738	CF
Firmicutes	Clostridia	Clostridiales	Clostridiaceae	Clostridium	perfringens	1.4968E-07	CF
Firmicutes	Clostridia	Clostridiales	diales_Family_XI_Incerta	Anaerococcus	unclassified	4.33E-05	CF
Firmicutes	Clostridia	Clostridiales	diales_Family_XI_Incerta	Finegoldia	magna	0.00121924	CF
Firmicutes	Negativicutes	Selenomonadales	Veillonellaceae	Megasphaera	micronuciformis	0.00010394	CF
Firmicutes	Negativicutes	Selenomonadales	Veillonellaceae	Veillonella	atypica	8.1747E-07	CF
Firmicutes	Negativicutes	Selenomonadales	Veillonellaceae	Veillonella	dispar	6.8452E-10	CF
Firmicutes	Negativicutes	Selenomonadales	Veillonellaceae	Veillonella	parvula	1.5372E-05	CF
Firmicutes	Negativicutes	Selenomonadales	Veillonellaceae	Veillonella	unclassified	0.00584231	CF
Proteobacteria	Gammaproteobacteria	Enterobacteriales	Enterobacteriaceae	Citrobacter	unclassified	0.00123319	CF
Proteobacteria	Gammaproteobacteria	Enterobacteriales	Enterobacteriaceae	Enterobacter	cloacae	0.00309602	CF
Proteobacteria	Gammaproteobacteria	Enterobacteriales	Enterobacteriaceae	Enterobacter	unclassified	0.00806746	CF
Proteobacteria	Gammaproteobacteria	Enterobacteriales	Enterobacteriaceae	Klebsiella	pneumoniae	2.5971E-06	CF
Proteobacteria	Gammaproteobacteria	Enterobacteriales	Enterobacteriaceae	Klebsiella	unclassified	0.01615305	CF
Proteobacteria	Gammaproteobacteria	Pseudomonadales	Pseudomonadaceae	Pseudomonas	unclassified	0.01860113	CF
Actinobacteria	Actinobacteria	Coriobacteriales	Coriobacteriaceae	Eggerthella	lenta	0.0076137	Control
Actinobacteria	Actinobacteria	Coriobacteriales	Coriobacteriaceae	Gordonibacter	pamelaeae	1.2765E-06	Control
Bacteroidetes	Bacteroidia	Bacteroidales	Bacteroidaceae	Bacteroides	caccae	3.8676E-05	Control
Bacteroidetes	Bacteroidia	Bacteroidales	Bacteroidaceae	Bacteroides	intestinalis	0.00194953	Control
Bacteroidetes	Bacteroidia	Bacteroidales	Bacteroidaceae	Bacteroides	ovatus	1.2127E-05	Control
Bacteroidetes	Bacteroidia	Bacteroidales	Bacteroidaceae	Bacteroides	unclassified	4.9762E-05	Control
Bacteroidetes	Bacteroidia	Bacteroidales	Bacteroidaceae	Bacteroides	vulgatus	2.9488E-06	Control
Bacteroidetes	Bacteroidia	Bacteroidales	Bacteroidaceae	Bacteroides	xylanisolvens	2.5962E-05	Control
Bacteroidetes	Bacteroidia	Bacteroidales	Rikenellaceae	Alistipes	putredinis	0.0002163	Control
Bacteroidetes	Bacteroidia	Bacteroidales	Rikenellaceae	Alistipes	shahii	0.00039538	Control
Firmicutes	Clostridia	Clostridiales	Clostridiaceae	Clostridium	asparagiforme	0.00010964	Control
Firmicutes	Clostridia	Clostridiales	Clostridiaceae	Clostridium	bartlettii	1.1194E-06	Control
Firmicutes	Clostridia	Clostridiales	Clostridiaceae	Clostridium	bolteae	0.00244916	Control
Firmicutes	Clostridia	Clostridiales	Clostridiaceae	Clostridium	cf	0.00036713	Control
Firmicutes	Clostridia	Clostridiales	Clostridiaceae	Clostridium	hathewayi	6.5408E-09	Control
Firmicutes	Clostridia	Clostridiales	Clostridiaceae	Clostridium	leptum	0.00261205	Control
Firmicutes	Clostridia	Clostridiales	Clostridiaceae	Clostridium	symbiosum	0.00309602	Control
Firmicutes	Clostridia	Clostridiales	Clostridiaceae	Pseudoflavonifractor	capillosus	6.70E-11	Control
Firmicutes	Clostridia	Clostridiales	Eubacteriaceae	Eubacterium	eligens	6.5593E-05	Control
Firmicutes	Clostridia	Clostridiales	Eubacteriaceae	Eubacterium	rectale	0.00172693	Control
Firmicutes	Clostridia	Clostridiales	Eubacteriaceae	Eubacterium	siraeum	0.00312883	Control
Firmicutes	Clostridia	Clostridiales	Eubacteriaceae	Eubacterium	ventriosum	0.00985228	Control
Firmicutes	Clostridia	Clostridiales	Lachnospiraceae	Anaerostipes	caccae	0.00087281	Control
Firmicutes	Clostridia	Clostridiales	Lachnospiraceae	Butyrivibrio	unclassified	9.0887E-05	Control
Firmicutes	Clostridia	Clostridiales	Lachnospiraceae	Roseburia	intestinalis	1.7538E-07	Control
Firmicutes	Clostridia	Clostridiales	Lachnospiraceae	Roseburia	inulinivorans	0.00047515	Control
Firmicutes	Clostridia	Clostridiales	Ruminococcaceae	Anaerotruncus	colihominis	3.3355E-07	Control
Firmicutes	Clostridia	Clostridiales	Ruminococcaceae	Faecalibacterium	cf	0.00068975	Control
Firmicutes	Clostridia	Clostridiales	Ruminococcaceae	Faecalibacterium	prausnitzii	8.847E-05	Control
Firmicutes	Clostridia	Clostridiales	Ruminococcaceae	Faecalibacterium	unclassified	0.00048685	Control
Firmicutes	Clostridia	Clostridiales	Ruminococcaceae	Ruminococcus	albus	0.01144006	Control
Firmicutes	Clostridia	Clostridiales	Ruminococcaceae	Ruminococcus	bromii	7.4882E-06	Control
Firmicutes	Clostridia	Clostridiales	Ruminococcaceae	Ruminococcus	lactaris	0.01531081	Control
Firmicutes	Clostridia	Clostridiales	Ruminococcaceae	Subdoligranulum	variabile	0.00019518	Control
Firmicutes	Erysipelotrichi	Erysipelotrichales	Erysipelotrichaceae	Coprobacillus	bacterium	0.00184527	Control
Firmicutes	Erysipelotrichi	Erysipelotrichales	Erysipelotrichaceae	Holdemania	filiformis	0.00234579	Control
Firmicutes	Negativicutes	Selenomonadales	Veillonellaceae	Dialister	invisus	0.00077629	Control
Proteobacteria	Deltaproteobacteria	Desulfovibrionales	Desulfovibrionaceae	Desulfovibrio	desulfuricans	2.6654E-07	Control
Verrucomicrobia	Verrucomicrobiae	Verrucomicrobiales	Verrucomicrobiaceae	Akkermansia	muciniphila	1.6932E-07	Control

* Wilcoxon rank-sum test p-value when comparing between CF and control samples; **The sample set in which the species is more abundant.

Table S4. Species found to be differentially abundant in CF or control samples, when excluding samples with antibiotic exposure in the prior 60 days

Phylum	Class	Order	Family	Genus	Species	p-value*	Abundant in**
Actinobacteria	Actinobacteria	Actinomycetales	Actinomycetaceae	Actinomyces	odontolyticus	0.00147238	CF
Firmicutes	Bacilli	Lactobacillales	Enterococcaceae	Enterococcus	faecalis	0.00223126	CF
Firmicutes	Bacilli	Lactobacillales	Enterococcaceae	Enterococcus	faecium	0.00108976	CF
Firmicutes	Bacilli	Lactobacillales	Lactobacillaceae	Lactobacillus	gasseri	0.017024	CF
Firmicutes	Bacilli	Lactobacillales	Streptococcaceae	Streptococcus	parasanguinis	9.7457E-05	CF
Firmicutes	Bacilli	Lactobacillales	Streptococcaceae	Streptococcus	vestibularis	0.01243332	CF
Firmicutes	Clostridia	Clostridiales	Clostridiaceae	Clostridium	perfringens	1.3561E-06	CF
Firmicutes	Clostridia	Clostridiales	diales_Family_XI_Incertae	Anaerococcus	unclassified	8.71E-05	CF
Firmicutes	Clostridia	Clostridiales	diales_Family_XI_Incertae	Finegoldia	magna	0.00082763	CF
Firmicutes	Negativicutes	Selenomonadales	Veillonellaceae	Megasphaera	micronuciformis	0.00011525	CF
Firmicutes	Negativicutes	Selenomonadales	Veillonellaceae	Veillonella	atypica	9.8374E-07	CF
Firmicutes	Negativicutes	Selenomonadales	Veillonellaceae	Veillonella	dispar	4.2328E-07	CF
Firmicutes	Negativicutes	Selenomonadales	Veillonellaceae	Veillonella	parvula	0.00138279	CF
Proteobacteria	Gammaproteobacteria	Enterobacteriales	Enterobacteriaceae	Escherichia	coli	8.7636E-07	CF
Proteobacteria	Gammaproteobacteria	Enterobacteriales	Enterobacteriaceae	Klebsiella	pneumoniae	0.00012184	CF
Actinobacteria	Actinobacteria	Coriobacteriales	Coriobacteriaceae	Eggerthella	lenta	0.00300859	Control
Actinobacteria	Actinobacteria	Coriobacteriales	Coriobacteriaceae	Gordonibacter	pamelaeae	8.7585E-06	Control
Bacteroidetes	Bacteroidia	Bacteroidales	Bacteroidaceae	Bacteroides	caccae	0.00089791	Control
Bacteroidetes	Bacteroidia	Bacteroidales	Bacteroidaceae	Bacteroides	intestinalis	0.00676331	Control
Bacteroidetes	Bacteroidia	Bacteroidales	Bacteroidaceae	Bacteroides	ovatus	0.00026034	Control
Bacteroidetes	Bacteroidia	Bacteroidales	Bacteroidaceae	Bacteroides	thetaiotaomicron	0.01723515	Control
Bacteroidetes	Bacteroidia	Bacteroidales	Bacteroidaceae	Bacteroides	unclassified	0.00072571	Control
Bacteroidetes	Bacteroidia	Bacteroidales	Bacteroidaceae	Bacteroides	vulgatus	4.3181E-06	Control
Bacteroidetes	Bacteroidia	Bacteroidales	Bacteroidaceae	Bacteroides	xylanisolvens	0.00042373	Control
Bacteroidetes	Bacteroidia	Bacteroidales	Rikenellaceae	Alistipes	putredinis	0.00026034	Control
Bacteroidetes	Bacteroidia	Bacteroidales	Rikenellaceae	Alistipes	shahii	0.00147238	Control
Firmicutes	Clostridia	Clostridiales	Clostridiaceae	Clostridium	asparagiforme	0.00019225	Control
Firmicutes	Clostridia	Clostridiales	Clostridiaceae	Clostridium	bartlettii	6.5053E-06	Control
Firmicutes	Clostridia	Clostridiales	Clostridiaceae	Clostridium	bolteae	2.8815E-05	Control
Firmicutes	Clostridia	Clostridiales	Clostridiaceae	Clostridium	cf	7.9223E-05	Control
Firmicutes	Clostridia	Clostridiales	Clostridiaceae	Clostridium	hathewayi	5.2836E-10	Control
Firmicutes	Clostridia	Clostridiales	Clostridiaceae	Clostridium	leptum	0.00774671	Control
Firmicutes	Clostridia	Clostridiales	Clostridiaceae	Clostridium	symbiosum	0.00233472	Control
Firmicutes	Clostridia	Clostridiales	Clostridiaceae	Pseudoflavonifractor	capillosus	7.70E-09	Control
Firmicutes	Clostridia	Clostridiales	Eubacteriaceae	Eubacterium	eligens	0.00091261	Control
Firmicutes	Clostridia	Clostridiales	Eubacteriaceae	Eubacterium	rectale	0.00658065	Control
Firmicutes	Clostridia	Clostridiales	Eubacteriaceae	Eubacterium	siraeum	0.006059	Control
Firmicutes	Clostridia	Clostridiales	Eubacteriaceae	Eubacterium	ventriosum	0.01107164	Control
Firmicutes	Clostridia	Clostridiales	Lachnospiraceae	Anaerostipes	caccae	0.00063555	Control
Firmicutes	Clostridia	Clostridiales	Lachnospiraceae	Butyrivibrio	unclassified	0.00064622	Control
Firmicutes	Clostridia	Clostridiales	Lachnospiraceae	Roseburia	intestinalis	1.1962E-07	Control
Firmicutes	Clostridia	Clostridiales	Lachnospiraceae	Roseburia	inulinivorans	0.00077511	Control
Firmicutes	Clostridia	Clostridiales	Ruminococcaceae	Anaerotruncus	colihominis	1.3561E-06	Control
Firmicutes	Clostridia	Clostridiales	Ruminococcaceae	Faecalibacterium	cf	0.00255492	Control
Firmicutes	Clostridia	Clostridiales	Ruminococcaceae	Faecalibacterium	prausnitzii	4.5367E-05	Control
Firmicutes	Clostridia	Clostridiales	Ruminococcaceae	Faecalibacterium	unclassified	0.00012642	Control
Firmicutes	Clostridia	Clostridiales	Ruminococcaceae	Ruminococcus	albus	0.01107164	Control
Firmicutes	Clostridia	Clostridiales	Ruminococcaceae	Ruminococcus	bromii	4.0341E-05	Control
Firmicutes	Clostridia	Clostridiales	Ruminococcaceae	Ruminococcus	obeum	0.01181081	Control
Firmicutes	Clostridia	Clostridiales	Ruminococcaceae	Subdoligranulum	variabile	0.00097369	Control
Firmicutes	Erysipelotrichi	Erysipelotrichales	Erysipelotrichaceae	Coprobacillus	bacterium	0.01243332	Control
Firmicutes	Erysipelotrichi	Erysipelotrichales	Erysipelotrichaceae	Holdemania	filiformis	0.00426343	Control
Firmicutes	Negativicutes	Selenomonadales	Veillonellaceae	Dialister	invisus	0.00121851	Control
Proteobacteria	Deltaproteobacteria	Desulfovibrionales	Desulfovibrionaceae	Bilophila	wadsworthia	0.01681512	Control
Proteobacteria	Deltaproteobacteria	Desulfovibrionales	Desulfovibrionaceae	Desulfovibrio	desulfuricans	4.4751E-08	Control
Verrucomicrobia	Verrucomicrobiae	Verrucomicrobiales	Verrucomicrobiaceae	Akkermansia	muciniphila	8.5628E-07	Control

* Wilcoxon rank-sum test p-value when comparing between CF and control samples; **The sample set in which the species is more abundant.

Table S5. Species found to be differentially abundant in CF or control samples, when excluding samples collected from concurrently breastfed subjects

Phylum	Class	Order	Family	Genus	Species	p-value*	Abundant in**
Actinobacteria	Actinobacteria	Actinomycetales	Actinomycetaceae	Actinomyces	odontolyticus	0.00088566	CF
Actinobacteria	Actinobacteria	Bifidobacteriales	Bifidobacteriaceae	Bifidobacterium	breve	0.00950464	CF
Actinobacteria	Actinobacteria	Coriobacteriales	Coriobacteriaceae	Collinsella	aerofaciens	0.00965103	CF
Firmicutes	Bacilli	Lactobacillales	Carnobacteriaceae	Granulicatella	adiacens	0.01530765	CF
Firmicutes	Bacilli	Lactobacillales	Streptococcaceae	Streptococcus	parasanguinis	0.00102908	CF
Firmicutes	Clostridia	Clostridiales	Clostridiaceae	Clostridium	difficile	0.00603455	CF
Firmicutes	Clostridia	Clostridiales	Clostridiaceae	Clostridium	perfringens	5.1116E-05	CF
Firmicutes	Clostridia	Clostridiales	diales_Family_XI_Incertae	Finegoldia	magna	0.00814614	CF
Firmicutes	Clostridia	Clostridiales	Clostridiales_uncl	Blautia	hansenii	0.00789629	CF
Firmicutes	Negativicutes	Selenomonadales	Veillonellaceae	Megasphaera	micronuciformis	9.93E-06	CF
Firmicutes	Negativicutes	Selenomonadales	Veillonellaceae	Veillonella	atypica	0.00011534	CF
Firmicutes	Negativicutes	Selenomonadales	Veillonellaceae	Veillonella	dispar	2.5345E-07	CF
Firmicutes	Negativicutes	Selenomonadales	Veillonellaceae	Veillonella	parvula	0.00205228	CF
Proteobacteria	Gammaproteobacteria	Enterobacteriales	Enterobacteriaceae	Citrobacter	unclassified	0.0032213	CF
Proteobacteria	Gammaproteobacteria	Enterobacteriales	Enterobacteriaceae	Enterobacter	cloacae	0.01465483	CF
Proteobacteria	Gammaproteobacteria	Enterobacteriales	Enterobacteriaceae	Escherichia	coli	1.1118E-08	CF
Proteobacteria	Gammaproteobacteria	Enterobacteriales	Enterobacteriaceae	Klebsiella	pneumoniae	4.2666E-05	CF
Proteobacteria	Gammaproteobacteria	Enterobacteriales	Enterobacteriaceae	Klebsiella	unclassified	0.00083683	CF
Proteobacteria	Gammaproteobacteria	Pseudomonadales	Pseudomonadaceae	Pseudomonas	unclassified	0.01265256	CF
Actinobacteria	Actinobacteria	Coriobacteriales	Coriobacteriaceae	Gordonibacter	pamelaeae	1.1486E-05	Control
Bacteroidetes	Bacteroidia	Bacteroidales	Bacteroidaceae	Bacteroides	caccae	0.00021283	Control
Bacteroidetes	Bacteroidia	Bacteroidales	Bacteroidaceae	Bacteroides	intestinalis	0.00707497	Control
Bacteroidetes	Bacteroidia	Bacteroidales	Bacteroidaceae	Bacteroides	ovatus	1.607E-05	Control
Bacteroidetes	Bacteroidia	Bacteroidales	Bacteroidaceae	Bacteroides	unclassified	0.00021283	Control
Bacteroidetes	Bacteroidia	Bacteroidales	Bacteroidaceae	Bacteroides	vulgatus	8.5543E-08	Control
Bacteroidetes	Bacteroidia	Bacteroidales	Bacteroidaceae	Bacteroides	xylanisolvens	0.00032058	Control
Bacteroidetes	Bacteroidia	Bacteroidales	Rikenellaceae	Alistipes	putredinis	0.00123855	Control
Bacteroidetes	Bacteroidia	Bacteroidales	Rikenellaceae	Alistipes	shahii	0.00106815	Control
Firmicutes	Clostridia	Clostridiales	Clostridiaceae	Clostridium	asparagiforme	0.00664087	Control
Firmicutes	Clostridia	Clostridiales	Clostridiaceae	Clostridium	bartlettii	0.00181231	Control
Firmicutes	Clostridia	Clostridiales	Clostridiaceae	Clostridium	cf	0.00480921	Control
Firmicutes	Clostridia	Clostridiales	Clostridiaceae	Clostridium	hathewayi	2.5669E-06	Control
Firmicutes	Clostridia	Clostridiales	Clostridiaceae	Clostridium	leptum	0.01122919	Control
Firmicutes	Clostridia	Clostridiales	Clostridiaceae	Pseudoflavonifractor	capillosus	9.07E-08	Control
Firmicutes	Clostridia	Clostridiales	Eubacteriaceae	Eubacterium	eligens	0.00091967	Control
Firmicutes	Clostridia	Clostridiales	Eubacteriaceae	Eubacterium	rectale	0.01228249	Control
Firmicutes	Clostridia	Clostridiales	Lachnospiraceae	Roseburia	intestinalis	5.4676E-05	Control
Firmicutes	Clostridia	Clostridiales	Ruminococcaceae	Anaerotruncus	colihominis	7.7749E-06	Control
Firmicutes	Clostridia	Clostridiales	Ruminococcaceae	Faecalibacterium	prausnitzii	0.01342241	Control
Firmicutes	Clostridia	Clostridiales	Ruminococcaceae	Ruminococcus	bromii	4.1708E-05	Control
Firmicutes	Clostridia	Clostridiales	Ruminococcaceae	Subdoligranulum	variabile	0.00205228	Control
Firmicutes	Erysipelotrichi	Erysipelotrichales	Erysipelotrichaceae	Coprobacillus	bacterium	0.00664087	Control
Firmicutes	Erysipelotrichi	Erysipelotrichales	Erysipelotrichaceae	Holdemania	filiformis	0.00244589	Control
Proteobacteria	Deltaproteobacteria	Desulfovibrionales	Desulfovibrionaceae	Desulfovibrio	desulfuricans	0.00017273	Control
Verrucomicrobia	Verrucomicrobiae	Verrucomicrobiales	Verrucomicrobiaceae	Akkermansia	muciniphila	1.2344E-06	Control

* Wilcoxon rank-sum test p-value when comparing between CF and control samples; **The sample set in which the species is more abundant.

Table S6. KEGG Pathways found to be significantly enriched in abundance in CF samples versus control samples.

Pathway	Median non-zero KOs*	p-value**
ko02060: Phosphotransferase system (PTS)	73	2.10E-12
ko00480: Glutathione metabolism	21	1.67E-08
ko00130: Ubiquinone and other terpenoid-quinone biosynthesis	22	4.09E-08
ko00650: Butanoate metabolism	60	3.83E-07
ko00640: Propanoate metabolism	53	1.01E-06
ko00627: Aminobenzoate degradation	27	1.60E-06
ko00410: beta-Alanine metabolism	23	1.83E-06
ko00380: Tryptophan metabolism	25	4.44E-06
ko00362: Benzoate degradation	39	5.02E-06
ko02010: ABC transporters	325	6.23E-06
ko03070: Bacterial secretion system	58	6.63E-06
ko02020: Two-component system	271	8.46E-06
ko00310: Lysine degradation	28	2.00E-05
ko00071: Fatty acid degradation	28	5.70E-04
ko00920: Sulfur metabolism	40	2.50E-03
ko00540: Lipopolysaccharide biosynthesis	30	2.91E-03
ko00790: Folate biosynthesis	27	5.50E-03

* Median number of KOs from the pathway that have are identified in each sample (see Methods); ** Wilcoxon rank-sum test p-value when comparing between CF and control samples.

Table S7. KEGG Pathways found to be significantly depleted in abundance in CF samples versus control samples.

Pathway	MedianNonZeroKOs*	p-value**
ko00520: Amino sugar and nucleotide sugar metabolism	93	1.64E-11
ko00250: Alanine, aspartate and glutamate metabolism	46	1.79E-11
ko00330: Arginine and proline metabolism	93	1.80E-10
ko00670: One carbon pool by folate	24	3.71E-09
ko03018: RNA degradation	31	1.38E-08
ko00190: Oxidative phosphorylation	87	1.67E-08
ko00061: Fatty acid biosynthesis	22	2.43E-08
ko04112: Cell cycle - Caulobacter	21	3.80E-08
ko00720: Carbon fixation pathways in prokaryotes	71	7.87E-08
ko00340: Histidine metabolism	30	8.16E-08
ko00730: Thiamine metabolism	20	8.16E-08
ko00710: Carbon fixation in photosynthetic organisms	29	1.72E-07
ko00500: Starch and sucrose metabolism	71	1.98E-07
ko00680: Methane metabolism	98	3.01E-07
ko03060: Protein export	23	4.24E-07
ko00620: Pyruvate metabolism	68	8.31E-07
ko00900: Terpenoid backbone biosynthesis	27	1.05E-06
ko03010: Ribosome	65	1.41E-06
ko00010: Glycolysis / Gluconeogenesis	71	2.86E-06
ko00550: Peptidoglycan biosynthesis	35	7.72E-06
ko00970: Aminoacyl-tRNA biosynthesis	38	1.04E-05
ko00240: Pyrimidine metabolism	86	1.08E-05
ko00030: Pentose phosphate pathway	44	1.11E-05
ko00770: Pantothenate and CoA biosynthesis	25	1.41E-05
ko03430: Mismatch repair	29	1.58E-05
ko00260: Glycine, serine and threonine metabolism	57	2.52E-05
ko03410: Base excision repair	21	7.32E-05
ko00300: Lysine biosynthesis	32	1.01E-04
ko03030: DNA replication	22	1.29E-04
ko00760: Nicotinate and nicotinamide metabolism	27	1.90E-04
ko00040: Pentose and glucuronate interconversions	42	2.11E-04
ko03440: Homologous recombination	33	5.05E-04
ko00400: Phenylalanine, tyrosine and tryptophan biosynthesis	49	1.62E-03
ko00230: Purine metabolism	125	0.00234579
ko00270: Cysteine and methionine metabolism	53	0.00234579
ko00020: Citrate cycle (TCA cycle)	47	0.00814539

* Median number of KOs from the pathway that have are identified in each sample (see Methods); ** Wilcoxon rank-sum test p-value when comparing between CF and control samples.

Table S8. KEGG Modules found to be significantly enriched in abundance in CF samples versus control samples.

Module	MedianNonZeroKOs*	p-value**
M00550: Ascorbate degradation, ascorbate => D-xylulose-5P	7	1.19E-13
M00117: Ubiquinone biosynthesis, prokaryotes, chorismate => ubiquinone	9	3.34E-12
M00529: Denitrification, nitrate => nitrogen	9	1.79E-11
M00324: Dipeptide transport system	5	5.88E-11
M00229: Arginine transport system	5	8.32E-11
M00334: Type VI secretion system	7	7.43E-10
M00331: Type II general secretion system	12	9.50E-10
M00545: Trans-cinnamate degradation, trans-cinnamate => 2-oxopent-4-enoate + fumarate	7	1.02E-08
M00530: Dissimilatory nitrate reduction, nitrate => ammonia	10	1.38E-08
M00095: C5 isoprenoid biosynthesis, mevalonate pathway	6	7.33E-08
M00546: Purine degradation, xanthine => urea	12	1.21E-07
M00080: Lipopolysaccharide biosynthesis, inner core => outer core => O-antigen	10	2.13E-07
M00121: Heme biosynthesis, glutamate => protoheme/siroheme	18	1.20E-06
M00194: Maltose/maltodextrin transport system	5	5.02E-06
M00440: Nickel transport system	5	5.02E-06
M00133: Polyamine biosynthesis, arginine => agmatine => putrescine => spermidine	6	1.36E-04
M00027: GABA (gamma-Aminobutyrate) shunt	5	1.55E-04
M00374: Dicarboxylate-hydroxybutyrate cycle	29	8.93E-04
M00429: Competence-related DNA transformation transporter	8	8.93E-04
M00307: Pyruvate oxidation, pyruvate => acetyl-CoA	9	1.38E-03
M00034: Methionine salvage pathway	12	6.45E-03
M00116: Menaquinone biosynthesis, chorismate => menaquinone	9	7.69E-03
M00239: Peptides/nickel transport system	5	3.46E-02
M00237: Branched-chain amino acid transport system	5	3.69E-02
M00176: Assimilatory sulfate reduction, sulfate => H2S	9	4.06E-02

* Median number of KOs from the pathway that have are identified in each sample (see Methods); ** Wilcoxon rank-sum test p-value when comparing between CF and control samples.

Table S9. KEGG Modules found to be significantly depleted in abundance in CF samples versus control samples.

Module	MedianNonZeroKOs*	p-value**
M00361: Nucleotide sugar biosynthesis, eukaryotes	8	3.64E-11
M00083: Fatty acid biosynthesis, elongation	12	5.34E-10
M00001: Glycolysis (Embden-Meyerhof pathway), glucose => pyruvate	22	2.60E-09
M00362: Nucleotide sugar biosynthesis, prokaryotes	11	2.81E-09
M00346: Formaldehyde assimilation, serine pathway	7	1.02E-08
M00051: Uridine monophosphate biosynthesis, glutamine (+ PRPP) => UMP	11	2.62E-08
M00359: Aminoacyl-tRNA biosynthesis, eukaryotes	21	3.04E-08
M00119: Pantothenate biosynthesis, valine/L-aspartate => pantothenate	6	5.89E-08
M00432: Leucine biosynthesis, 2-oxoisovalerate => 2-oxoisocaproate	5	9.09E-08
M00096: C5 isoprenoid biosynthesis, non-mevalonate pathway	9	1.01E-07
M00028: Ornithine biosynthesis, glutamate => ornithine	11	1.66E-07
M00002: Glycolysis, core module involving three-carbon compounds	10	2.13E-07
M00572: Pimeloyl-ACP biosynthesis, BioC-BioH pathway, malonyl-ACP => pimeloyl-ACP	8	3.83E-07
M00360: Aminoacyl-tRNA biosynthesis, prokaryotes	28	4.10E-07
M00049: Adenine ribonucleotide biosynthesis, IMP => ADP,ATP	5	4.54E-07
M00010: Citrate cycle, first carbon oxidation, oxaloacetate => 2-oxoglutarate	5	5.20E-07
M00127: Thiamine biosynthesis, AIR => thiamine-P/thiamine-2P	8	6.80E-07
M00173: Reductive citrate cycle (Arnon-Buchanan cycle)	33	7.78E-07
M00115: NAD biosynthesis, aspartate => NAD	7	8.04E-07
M00179: Ribosome, archaea	34	1.01E-06
M00048: Inosine monophosphate biosynthesis, PRPP + glutamine => IMP	16	1.12E-06
M00178: Ribosome, bacteria	55	1.36E-06
M00159: V-type ATPase, prokaryotes	8	1.45E-06
M00029: Urea cycle	5	1.71E-06
M00050: Guanine ribonucleotide biosynthesis IMP => GDP,GTP	5	2.14E-06
M00345: Formaldehyde assimilation, ribulose monophosphate pathway	6	2.68E-06
M00120: Coenzyme A biosynthesis, pantothenate => CoA	9	3.45E-06
M00021: Cysteine biosynthesis, serine => cysteine	5	3.79E-06
M00026: Histidine biosynthesis, PRPP => histidine	15	4.30E-06
M00125: Riboflavin biosynthesis, GTP => riboflavin/FMN/FAD	9	4.44E-06
M00036: Leucine degradation, leucine => acetoacetate + acetyl-CoA	11	6.63E-06
M00003: Gluconeogenesis, oxaloacetate => fructose-6P	12	7.49E-06
M00527: Lysine biosynthesis, DAP aminotransferase pathway, aspartate => lysine	9	9.26E-06
M00526: Lysine biosynthesis, DAP dehydrogenase pathway, aspartate => lysine	8	1.68E-05
M00023: Tryptophan biosynthesis, chorismate => tryptophan	13	1.73E-05
M00565: Trehalose biosynthesis, D-glucose 1P => trehalose	9	1.73E-05
M00157: F-type ATPase, prokaryotes and chloroplasts	8	1.89E-05
M00082: Fatty acid biosynthesis, initiation	11	2.06E-05
M00335: Sec (secretion) system	13	3.26E-05
M00549: Nucleotide sugar biosynthesis, glucose => UDP-glucose	6	8.16E-05
M00596: Dissimilatory sulfate reduction, sulfate => H2S	5	8.61E-05
M00019: Valine/isoleucine biosynthesis, pyruvate => valine / 2-oxobutanoate => isoleucine	6	1.36E-04
M00007: Pentose phosphate pathway, non-oxidative phase, fructose 6P => ribose 5P	5	1.95E-04
M00525: Lysine biosynthesis, acetyl-DAP pathway, aspartate => lysine	9	0.000316195
M00061: Uronic acid metabolism	7	0.000612258
M00377: Reductive acetyl-CoA pathway (Wood-Ljungdahl pathway)	10	0.000627077
M00333: Type IV secretion system	10	0.000794763
M00373: Ethylmalonyl pathway	7	0.001003298
M00570: Isoleucine biosynthesis, threonine => 2-oxobutanoate => isoleucine	7	0.001151645
M00165: Reductive pentose phosphate cycle (Calvin cycle)	14	0.001545094
M00439: Oligopeptide transport system	5	0.001805017
M00122: Cobalamin biosynthesis, cobinamide => cobalamin	7	0.001928253
M00183: RNA polymerase, bacteria	6	0.001928253

M00018: Threonine biosynthesis, aspartate => homoserine => threonine	9	0.001971019
M00016: Lysine biosynthesis, succinyl-DAP pathway, aspartate => lysine	12	0.002396963
M00022: Shikimate pathway, phosphoenolpyruvate + erythrose-4P => chorismate	13	0.002668513
M00200: Putative sorbitol/mannitol transport system	5	0.003660459
M00260: DNA polymerase III complex, bacteria	10	0.004316969
M00004: Pentose phosphate pathway (Pentose phosphate cycle)	12	0.006975903
M00532: Photorespiration	12	0.009668298
M00582: Energy-coupling factor transport system	10	0.016442705
M00491: Putative arabinogalactan oligomer transport system	5	0.020290967
M00012: Glyoxylate cycle	7	0.022113209
M00506: CheA-CheYBV (chemotaxis) two-component regulatory system	5	0.023273549
M00356: Methanogenesis, methanol => methane	5	0.029901227

* Median number of KOs from the pathway that have are identified in each sample (see Methods); ** Wilcoxon rank-sum test p-value when comparing between CF and control samples.

Table S10. Results for subsets of samples excluding those collected after antibiotic exposure or during breastfeeding.

Result	All samples	Exclude ABx 30	Exclude ABx 60	Exclude breastfed	Only one sample per individual from age 0-365 days
Number of samples	52 CF vs. 52 non-CF	39 CF vs. 50 non-CF	35 CF vs. 49 non-CF	32 CF vs. 44 non-CF	10 CF vs. 6 non-CF
Pathways enriched/depleted in CF samples	17/36 (5% FDR)	16/32 (5% FDR)	15/30 (5% FDR)	16/29 (5% FDR)	9/26 (10% FDR)
Modules enriched/depleted in CF samples	25/65 (5% FDR)	21/58 (5% FDR)	20/53 (5% FDR)	20/43 (5% FDR)	8/3 (10% FDR)
Fatty acid degradation enriched in CF	Yes	Yes	Yes	No	No
Fatty acid biosynthesis depleted in CF	Yes	Yes	Yes	Yes	Yes (10% FDR)
Fatty acid biosynthesis modules depleted in CF	M00083, M00082	M00083, M00082	M00083, M00082	M00083, M00082	M00083 (10% FDR)
Butyrate catabolism module enriched in CF	P=8.04E-07	P=0.0008	P=0.004	P=0.0019	P=0.05
Butyrate non-catabolism module enriched in CF	P=1.32E-04	P=0.0005	P=0.00057	P=0.0068	P=0.51
Pronounced increase in the abundance of the butyrate catabolic module in CF compared to the non-catabolic module	1.95-fold vs. 1.07-fold	1.71-fold vs. 1.06-fold	1.66-fold vs. 1.06-fold	1.71-fold vs. 1.05-fold	2.46-fold vs. 1-fold
Propionate catabolism module enriched in CF	P=0.00023	P=0.0023	P=0.0093	P=0.042	P=0.039
Pronounced increase in the abundance of the propionate catabolic module in CF compared to the non-catabolic module	1.47-fold vs. 1.02-fold	1.42-fold vs. 1.01-fold	1.38-fold vs. 1.01-fold	1.28-fold vs. 1.04-fold	1.69-fold vs. 1-fold
Significant positive correlation between the abundance of the butyrate and propionate catabolism modules and fecal **fat content**	r=0.607 (P<1.0E-04) and r=0.468 (P<1.0E-04)	r=0.67 (P<1.0E-04) and r=0.53 (P<1.0E-04)	r=0.67 (P<1.0E-04) and r=0.51 (P<1.0E-04)	r=0.53 (P<1.0E-04) and r=0.26 (P=0.0294)	r=0.57 (P=0.0207) and r=0.61 (P=0.0134)
Significant positive correlation between the abundance of the butyrate and propionate catabolism modules and fecal **inflammation index**	r=0.5 (P<1.0E-04) and r=0.445 (P<1.0E-04)	r=0.58 (P<1.0E-04) and r=0.52 (P<1.0E-04)	r=0.53 (P<1.0E-04) and r=0.49 (P=0.0001)	r=0.43 (P=0.001) and r=0.38 (P=0.002)	r=0.538 (P=0.0293) and r=0.527 (P=0.0334)

Table S11. List of KEGG orthology groups (KOs) that have evidence in the literature to be involved in the catabolism of butyrate.

Name	Enzyme	KOs	Reference
bcd	1.3.8.1	K00248	Müller N. *et al* ., *Environ Microbiol Rep* . 2(4), 489-99 (2010). And see also: Ikeda Y. et al., J Biol Chem. 260(2), 1311-25 (1985).
atoD, atoA	2.8.3.8	K01034, K01035	Janssen P.H. and Bernhard S., *J Bacteriol* . 177(13), 3870-2 (1995).
ACSM	6.2.1.2	K01896	
echA, ECHS1, EHHADH, HADHA	4.2.1.17	K01692, K07511, K07514, K07515	
fadJ, fadB, HADH	1.1.1.35	K01782, K01825, K00022	
phbB	1.1.1.36	K00023	
atoB	2.3.1.9	K00626	
bdh	1.1.1.30	K00019	

Table S12. List of KEGG orthology groups (KOs) that have evidence in the literature to be involved in the catabolism of propionate.

Name	Enzyme	KOs	Reference
prpB	4.1.3.30	K03417	Suvorova I.A. *et al* , *J Bacteriol* . 194(12), 3234-40 (2012).
prpC	2.3.3.5	K01659	
prpE	6.2.1.17	K01908	
pccA, pccB	6.4.1.3	K01964, K01965, K01966, K15036	
MCEE	5.1.99.1	K05606	
MUT, mcmA1, mcmA2	5.4.99.2	K01847, K01848, K01849, K11942	
acnB	4.2.1.3	K01682	
prpD	4.2.1.79	K01720	
mmcD	4.1.1.41	K11264	Kosaka T. *et al* ., *J Bacteriol* . 188(1), 202-10 (2006).
LSC1, LSC2, sucD, sucC	6.2.1.5	K01899, K01900, K01902, K01903	

Table S13. Comparison of KEGG pathways with manual separation to catabolism and non-catabolism modules.

Pathway / module	# KOs*	Mean relative abundance in CF	Mean relative abundance in control	p-value**	fold-ratio CF/control
Butyrate metabolism	70	0.0097	0.0084	3.83E-07	1.14
Butyrate catabolism enzymes	14	0.0010715	0.00055017	8.04E-07	1.95
Butyrate non-catabolism enzymes	50	0.007785	0.0072958	1.32E-04	1.07
propionate metabolism	66	0.0089	0.0081	1.01E-06	1.10
propionate catabolism enzymes	19	0.0018663	0.0012687	2.34E-04	1.47
propionate non-catabolism enzymes	43	0.0067485	0.006593	5.67E-01	1.02

* The number of KOs that are included in the pathway/module (see Methods); ** Wilcoxon rank-sum test p-value when comparing between CF and control samples.

Table S14. Comparison of KEGG pathways with manual separation to catabolism and non-catabolism modules.

		Inflammation index (calprotein)			Fat content		
**Pathway / module**	**# KOs***	**Correlation****	**p-value*****	**Passes Bonferonni**	**Correlation****	**p-value*****	**Passes Bonferonni**
Butyrate metabolism	70	0.292	0.0015	yes	0.418	< 1.0E-04	yes
Butyrate catabolism enzymes	14	0.501	< 1.0E-04	yes	0.607	< 1.0E-04	yes
Butyrate non-catabolism enzymes	50	0.246	0.0201	no	0.407	0.0005	yes
propionate metabolism	66	0.31	0.0009	yes	0.419	< 1.0E-04	yes
propionate catabolism enzymes	19	0.445	< 1.0E-04	yes	0.468	< 1.0E-04	yes
propionate non-catabolism enzymes	43	-0.0163	0.536	no	0.0695	0.263	no

* The number of KOs that are included in the pathway/module; ** Pearson's correlation of abundance profile with inflammation or fat content; *** Pearson's correlation significance test p-value using 10,000 permutations
